# Fine-Scale analysis of both wild and cultivated horned galls provides insight into their quality differentiation

**DOI:** 10.1186/s12870-023-04442-1

**Published:** 2023-09-14

**Authors:** Xufang Tian, Ziyang Sang, Zhaohui Lan, Wei Liu, Ying Feng, Juan Hu, Faju Chen, Yifei Liu

**Affiliations:** 1 College of Pharmacy, Hubei University of Chinese Medicine, Wuhan, 430065 People’s Republic of China; 2 Forestry Science Research Institute of Wufeng County, Yichang, 443400 People’s Republic of China; 3 Biotechnology Research Center, China Three Gorges University, Yichang, 443002 People’s Republic of China

**Keywords:** Galla chinensis, Horned gall, *Rhus chinensis*, *Schlechtendalia chinensis*, Quality degradation, Genetic diversity

## Abstract

**Background:**

Galla chinensis is a traditional Chinese medicine (TCM) produced due to the interaction between the Fordinae aphids and the *Rhus* plant species. Horned galls with high tannin content are the most widely cultivated gall type, and Wufeng county of Hubei province in China is the center of cultivation. However, long-term artificial cultivation and domestication of horned galls to meet the increasing production demand have led to quality degradation. Understanding the reasons underlying quality degradation is urgent for horned gall production and application. The present study used a combination of metabolic, genetic, and ecological analyses to investigate the quality and genetic differentiation of the horned galls under long-term domestication as well as the potential relationships between them.

**Results:**

Analysis of gallic acid content and other three phenotypic traits (fresh weight, gall size, and wall thickness) revealed quality differentiation of horned galls collected from five locations in Wufeng, in which the cultivated samples from Wang Jiaping (WJP) showed the highest degradation. Genetic differentiation between the cultivated and wild *Rhus chinensis* trees in WJP, and between WJP and the other populations was detected based on SSR molecular markers, however, no significant difference in genetic structure was seen for the aphid populations. Among the various ecological factors examined, temperature was identified as the primary one affecting the quality of horned galls.

**Conclusions:**

Both genetic and ecological factors caused quality differentiation of horned galls. The collection of diverse germplasm of host trees and aphids will help reduce the quality degradation of horned galls in Wufeng.

**Supplementary Information:**

The online version contains supplementary material available at 10.1186/s12870-023-04442-1.

## Background

Galla chinensis is a kind of insect galls formed by the parasitic aphids of Fordinae on the leaves of *Rhus* plants. According to the shape, Galla chinensis is divided into three types: horned galls, gallnuts, and flower-like galls (See Supplementary Fig. 1, Additional File 1). Among these, the horned galls are the most widely generated galls in field with relatively high yield and high tannin content [1, 2]. Galla chinensis has been used as traditional Chinese medicine (TCM) since the Tang Dynasty due to its natural pharmaceutical values, such as anti-diarrheal [3], antibacterial [4], anti-inflammatory [5], and anti-anaphylactic properties [6]. Due to its high tannin content, Galla chinensis also plays a crucial role in industries on leatherworking, food and feed additives, textile printing, dyes, inks, and mineral separation [7]. Asia is the major producer of Galla chinensis, and the products from China (also known as Chinese gallnuts) alone account for about 95% of the global yields [2].

Galla chinensis is produced via a complex interaction between the gall-inducing aphids and the summer and winter hosts. For example, the horned galls are induced by the fundatrix of *Schlechtendalia chinensis* parasitizing on the leaf wings of their summer host, *Rhus chinensis*, in early May. Then, a fundatrix is imprisoned in an initial gall and reproduces parthenogenetically for several generations during May to October, during which the aphid population and the gall size increase exponentially When the number of aphids and the size of galls reach a certain level, the mature gall cracks, and the winged aphids migrate to their winter host mosses, wherein they live through the winter. As spring arrives, the winged sexuparae fly back to *R. chinensis* to reproduce sexual males and females, then sexual aphids mate with each other and breed into wingless fundatrix, which suck the sap of leaf wings, initiate a horned gall, and start their new life cycle [7–10]. Both the host trees and the aphids are key factors contributing to the yield and quality of Gall chinensis.

Wufeng County in the Hubei Province of China is famous for the artificial production of horned galls. In addition to the potential domestication and cultivation center of Wang Jiaping (WJP), more than half of the villages in Wufeng, such as Bai Nianguan (BNG), Bai Luzhuang (BLZ), Huang Liangping (HLP), and Huo Shan (HS), produce horned galls. In WJP, the cultivation of the host plant *R. chinensis* and artificial propagation of the parasitic aphid *S. chinensis* started several decades ago, while those in the remaining areas of BNG, BLZ, HLP, and HS have just begun. The increasing market demands have led to a rise in horned gall production; however, the quality of galls has been degrading due to the long-term monoculture. In general, cultivars show a lower genetic diversity [11–13], and the active ingredient content of cultivars is possibly unstable and fluctuating when catering to the market demand of high yield [14–16]. Therefore, the effects of long-term artificial cultivation on horned galls need to be investigated.

Genetic and environmental factors influence the synthesis and accumulation of chemical ingredients in diverse medicinal plants [17–23]. Studies have demonstrated that temperature, light, and precipitation are crucial environmental factors influencing the biosynthesis of plant secondary metabolites [20]. High temperature stress increased the production of active ingredients, such as phenolics, in *Astragalus compactus* [24], whereas similar environmental conditions reduced metabolites in *chrysanthemum* [25]. Meanwhile, appropriate sunlight intensity is critical for the synthesis of chemical ingredients in plants, such as alkaloids, hexadecenoic acid, flavonoids, phenolic acids, and spermine [26–28]. However, the impact of environmental or climatic factors on the quality of horned galls is less investigated.

The previous studies on Galla chinensis primarily focused on illustrating the formation of galls and explored the host plant–parasitic aphid interaction mechanism [7–9, 29]. However, the differentiation of horned gall quality under the current large-scale cultivation pattern and the underlying mechanisms remain unknown. Therefore, the present study analyzed the phenotypic variations among the wild and cultivated horned galls collected from five locations abovementioned in Wufeng. Besides, the genetic diversity of both the *R. chinensis* host trees and the *S. chinensis* aphids, and the environmental factors influencing the production of horned galls were investigated to elucidate the potential mechanism underlying the degradation of cultivated horned galls. Our results will assist in improving the cultivation and applications of Galla chinensis.

## Results

### Determination of the gallic acid content and other phenotypic traits

**Fig. 1:**
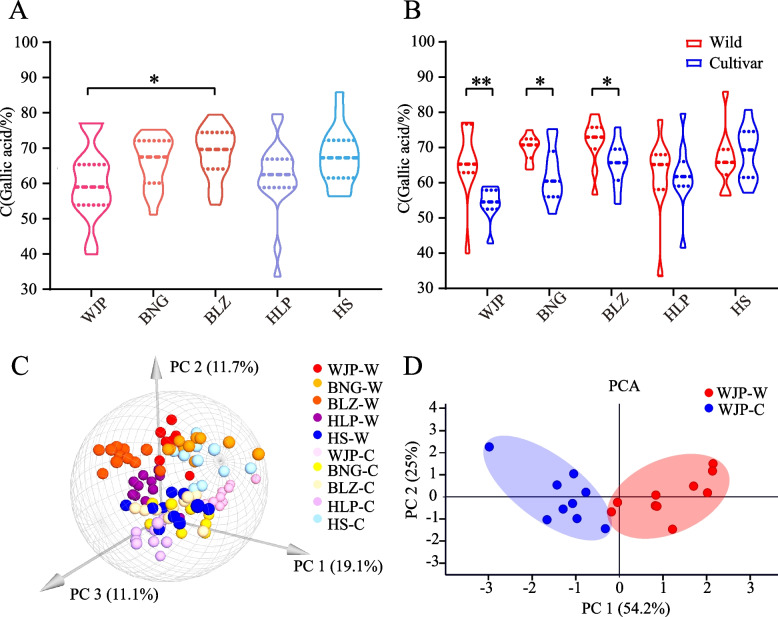
Analysis of the gallic acid content and three other phenotypic traits of horned galls collected from Wufeng. (**A**) the gallic acid contents of the horned galls from five locations (WJP, BNG, BLZ, HLP, and HS represent the population from Wang Jiaping, Bai Nianguan, Bai Luzhuang, Huang Liangping, and Huo Shan, respectively). (**B**) the gallic acid contents in wild and cultivated horned galls in each location. (**C**) Principal Component Analysis (PCA) analysis of the phenotypic traits of all horned gall accessions. (**D**) PCA analysis of the phenotypic traits for wild and cultivated horned galls in WJP. (*, *P* < 0.05; **, *P* < 0.01)

The average gallic acid content in the samples from five locations ranged from 60.35% (WJP) to 68.66% (BLZ), and the content in WJP samples was significantly lower than that in BLZ (*P* < 0.05) (Fig. 1A, Supplementary Table 1, Additional File 1). The comparison of the samples from each location, showed that the content of gallic acid in the wild populations (67.26%) was generally higher than that in the cultivated populations (62.58%) (See Supplementary Table 1, Additional File 1), especially in WJP, BNG, and BLZ (Fig. 1B). Thus, the analysis of Galla chinensis based on the gallic acid content indicated a potential quality degradation in cultivated populations in WJP, BNG and BLZ (Fig. 1B, Supplementary Table 1, Additional File 1).

The analysis of three other phenotypic traits also revealed significant differences between wild and cultivated horned galls. The gall fresh weight and size in the wild populations were significantly lower than in the cultivated populations, especially in WJP, BLZ, and HLP (See Supplementary Fig. 2F and H, Additional File 1). However, no obvious difference was detected in wall thickness between the wild and cultivated populations (See Supplementary Fig. 2J, Additional File 1). Moreover, there were significant differences in the range of variation between the wild and cultivated individuals. By analyzing the above four phenotypic indices related to the quality of horned galls, we can see a trend that the variation range of wild populations was wider than that of cultivated populations (See Supplementary Fig. 2A, B, and C, Additional File 1), such as gallic acid content (wild: 33.60%—85.90%; cultivated: 41.55%—80.71%), fresh weight (wild: 1.80 g—21.83 g; cultivated: 5.65 g—24.30 g), and gall size (wild: 2.67 mL—43.33 mL; cultivated: 9.00 mL—38.33 mL), revealing a greater divergence among the wild individuals. These observations suggest significant differences of phenotypes presented between wild and cultivated populations.

PCA performed using a combination of data on fresh weight, gall size, wall thickness and gallic acid content showed that the characteristics of the horned galls from each population were different, although partially overlapped (Fig. 1C). Moreover, the phenotypes between the wild and cultivated horned galls revealed different levels of differentiation across populations (Fig. 1D and Supplementary Fig. 3, Additional File 1). For example, a clear phenotypic differentiation was found between the wild and cultivated samples from WJP (Fig. 1C and 1D), suggesting potential quality degradation in the cultivated horned galls.

### Characterization of SSR markers

**Fig. 2:**
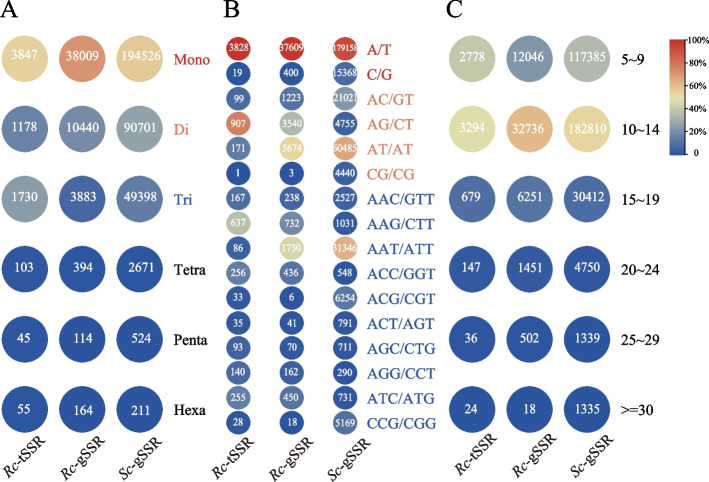
The distribution characters of SSRs. Abundance of each SSR type (**A**), frequency distribution of principal type in SSR motifs (**B**), and distribution of repeats number (**C**) were revealed. *Rc*-tSSR means the transcriptome derived SSRs of *Rhus chinensis*, *Rc*-gSSR means the genomic SSRs of *R. chinensis*, and the *Sc*-gSSR means the genomic SSRs of *Schlechtendalia chinensis*

Due to the lack of availability of genome information on *R. chinensis*, the transcriptome data based on RNA-seq and reduced-representation genome data based on ddRAD-seq were used to develop the transcriptomic SSRs (*Rc*-tSSR) and the genomic SSRs (*Rc*-gSSR), respectively. The clean reads obtained from RNA-seq were de novo assembled into 34,976 unigenes, which were further functionally annotated (See Supplementary Fig. 4, Additional File 1). While the dd-RAD sequences were assembled into 69,134 contigs and 3,438,148 unitigs (Supplementary Table 3, Additional File 1). Thus, 6,958 *Rc*-tSSRs and 51,937 *Rc*-gSSRs were identified (Supplementary Table 3, Additional File 1). The *Rc*-tSSRs and *Rc*-gSSRs of the host showed similar repeat types with a majority of mononucleotide (55.28%, 71.71%), followed by dinucleotides (16.93%, 19.70%) and trinucleotides (24.86%, 14.61%) (Fig. 2A). The A/T repeat type was predominant (99.51%, 98.95%) among the mononucleotides in both transcriptome and genome data. In addition, the AG/CT (76.99%) and AT/AT (54.35%) were the primary dinucleotide repeat types, and AAG/CTT (36.82%) and AAT/ATT (44.55%) were the main trinucleotides among transcriptomic and genomic SSRs, respectively (Fig. 2B). Besides, the majority of *R. chinensis* SSRs of both kinds had 10 to 14 repeats (Fig. 2C).

For *S. chinensis*, 202 sequences containing 338,031 SSR loci (*Sc*-gSSR) were detected from the genome (Supplementary Table 3, Additional File 1). This collection of SSR loci had 194,526 mononucleotide repeats (57.54%), 90,701 dinucleotide repeats (26.83%), and 49,398 trinucleotide repeats (14.61%) (Fig. 2A). Among these, A/T type was the most frequent mononucleotide repeat (92.10%), AT/AT type was the predominant dinucleotide repeat (66.69%), and AAT/ATT type was the predominant trinucleotide repeat (63.46%) (Fig. 2B). Among these, 54.08% of the SSRs had 10 to 14 repeats (Fig. 2C).

### Validations of SSR markers and analysis of genetic diversity

For *R. chinensis*, 23 pairs of SSR markers, including 12 *Rc*-tSSRs and 11 *Rc*-gSSRs that passed two rounds of primer selection, were retained for genetic analysis (Supplementary Table 4, Additional File 1). A total of 151 alleles were detected in 102 samples by applying these SSRs. The average *N*_A_, the mean *PIC*, and the mean *N*_E_ values per marker were 6.57, 0.50 and 2.52, respectively. Besides, no significant difference in genetic diversity was observed between the two types of SSR markers (Supplementary Table 4, Additional File 1). For *S. chinensis*, nine pairs of high-quality SSR primers were finally obtained, with which 85 alleles were amplified from 102 aphid samples (Supplementary Table 4, Additional File 1). The average *N*_A_ was 9.44 alleles per locus, the mean *PIC* value was 0.67, and the mean *N*_E_ value was 4.23 (Supplementary Table 4, Additional File 1).

The genetic diversity of both host trees and aphids was further analyzed (See Supplementary Fig. 5, Additional File 1). For the host trees, the genetic diversity of the cultivars was slightly lower level than those of the wild individuals in WJP, while no significant difference was observed between the wild and cultivated samples from the other locations (See Supplementary Fig. 5, Additional File 1). Similarly, the genetic diversity of aphids from the cultivated samples of WJP was slightly lower than that from the wild samples. At the same time, no significant difference was found in the populations of other locations (See Supplementary Fig. 5, Additional File 1). Besides, the wild aphids in WJP (WJP-W) had the highest level of genetic diversity, while those from BLZ (BLZ-W) had the most impoverished genetic diversity (See Supplementary Fig. 5, Additional File 1).

### Population structure of both host trees and aphids

**Fig. 3:**
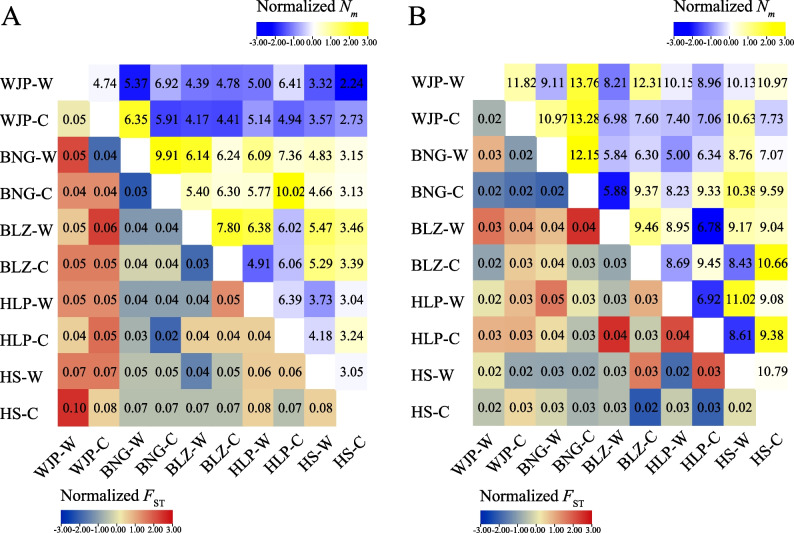
Pairwise genetic differentiation coefficient (*F*_ST_, below diagonal) and gene flow (*N*_*m*_, above diagonal) among ten populations of *R. chinensis* (**A**) and *S. chinensis* (**B**)

**Fig. 4:**
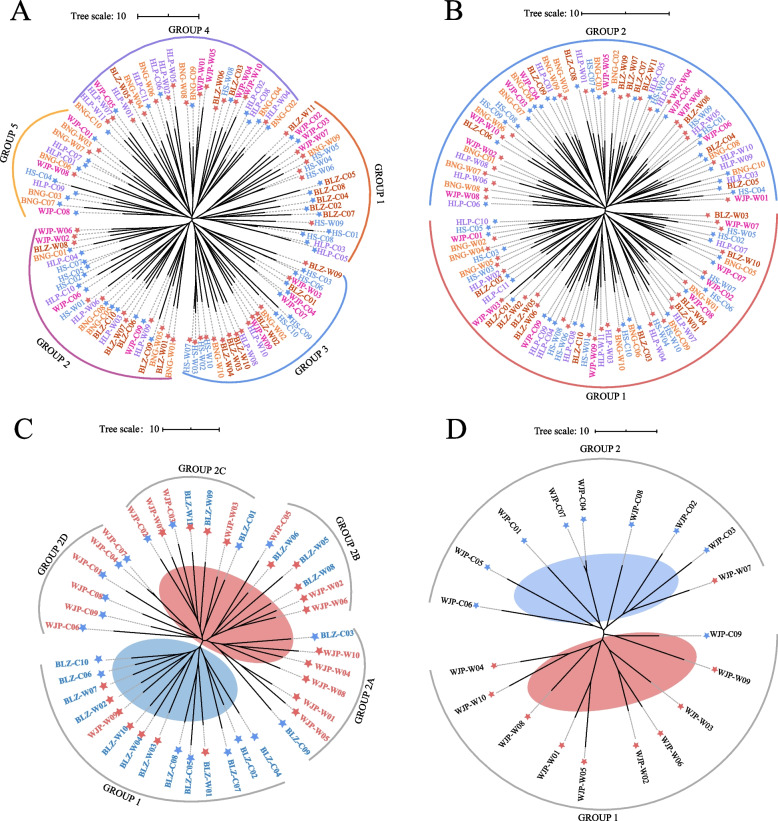
Neighbour-Joining (NJ) trees of *R. chinensis* and *S. chinensis*. The NJ tree of *R. chinensis* samples (**A**) and *S. chinensis* samples (**B**). (**C**) NJ tree of *R. chinensis* individuals from WJP and BLZ. (**D**) NJ tree of *R. chinensis* individuals in WJP. The leave labels were coloured to represent the geographic populations, the red stars represent the wild individuals while the blue stars represent the cultivars

The overall genetic differentiation between *R. chinensis* populations was weak except for the WJP samples, which revealed a relatively high *F*_ST_ value but a low level of gene flow (*N*_*m*_) compared with the other populations (Fig. 3A). The NJ tree divided all *R. chinensis* individuals into five groups (Fig. 4A), which was further supported by the STRUCTURE analysis (See Supplementary Fig. 6A, Additional File 1). However, this classification based on genetic distance was not consistent with the geographical distribution of populations, suggesting commonly genetic background or wide genetic admixture between population samples. The AMOVA analysis also clarified that most genetic variation (94%) occurred within populations rather than between populations (6%) (See Supplementary Table 5, Additional File 1).

The NJ tree (Fig. 4B) and STRUCTURE analysis (See Supplementary Fig. 6B, Additional File 1) showed two major genetic groups among the *S. chinensis* aphid samples. The populations based on the geographical location did not correspond well to the two genetic groups, consistent with the high gene flow (*N*_*m*_ > 1) and extremely low genetic differentiation (*F*_ST_ < 0.05) between populations (Fig. 3B). The AMOVA analysis showed no genetic differentiation among the aphid populations (See Supplementary Table 5, Additional File 1).

Further, to trace the causes of quality degradation in the cultivated horned galls, we analyzed the samples of each location in detail. Since no genetic differentiation was detected between the aphid populations, we analyzed only the *R. chinensis* populations. The previous analysis showed that the gallic acid content and other phenotypic traits of the galls from WJP significantly differed from those of BLZ (Fig. 1A and C). Therefore, we first compared the WJP and BLZ populations and found clear genetic differentiation between them based on the NJ tree (Fig. 4C), STRUCTURE analysis (See Supplementary Fig. 6C, Additional File 1), and PCA analysis (See Supplementary Fig. 6D, Additional File 1). In the NJ tree, most BLZ individuals were in GROUP 1, while most accessions from WJP were in GROUP 2. GROUP 2 was further divided into four subgroups: GROUP 2A mainly represented the wild WJP individuals, GROUP 2B had the wild accessions from both the WJP and BLZ locations, GROUP 2C was a combination of both the wild and cultivated *R. chinensis* trees from WJP and BLZ, and GROUP 2D included the cultivated samples of WJP (Fig. 4C). In addition to the observed genetic differentiation between BLZ and WJP, we further conducted analysis on the WJP population individually because the most severe quality degradation was detected for the cultivated horned galls of this location (Fig. 1B). Clearly, the WJP wild and cultivated samples revealed significant genetic differentiation (Fig. 4D, See Supplementary Figs. 6E, F, and 6, Additional File 1).

### Variation in ecological factors among the sampling locations

**Fig. 5:**
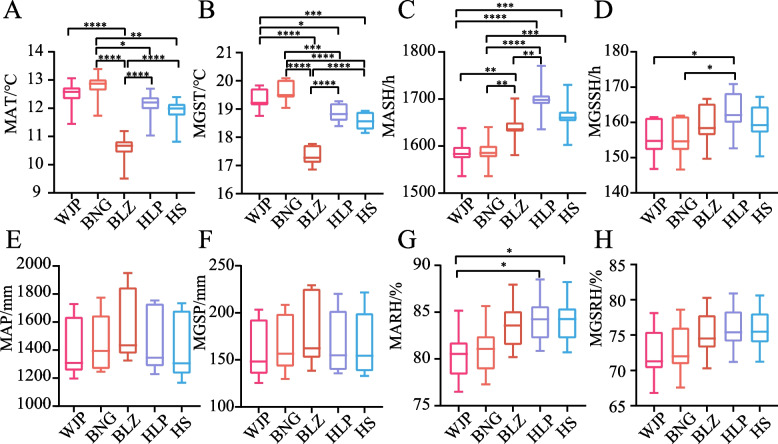
Comparative analysis of represented ecological factors among five locations over a decade (2012 to 2021). Key ecological factors include (**A**) mean annual temperature (MAT), (**B**) mean growing-season temperature (MGST), (**C**) mean annual sunshine hours (MASH), (**D**) mean growing-season sunshine hours (MGSSH), (**E**) mean annual precipitation (MAP), (**F**) mean growing-season precipitation (MGSP), (**G**) mean annual relative humidity (MARH), and (**H**) mean growing-season relative humidity (MGSRH). (*, *P* < 0.05; **, *P* < 0.01; ***, *P* < 0.001; ****, *P* < 0.0001)

Ecological factors significantly affect plant growth characteristics [14, 20–23, 30]. Therefore, to investigate the relationship between the observed quality degradation in the horned galls and the ecological conditions of the location, data on eight climatic factors for the past decade (2012 to 2021) were extracted. The temperature and the sunshine duration were significantly different among the five locations but the precipitation and the relative humidity were not (Fig. 5). The annual average temperature and the average temperature in growing season in WJP were significantly different from those in BLZ (*P* < 0.0001) (Fig. 5A and B). Similarly, the average annual temperature and mean growing-season temperature in BLZ significantly differed from the other places (Fig. 5A and B). These observations collectively suggest the potential influence of climatic factors such as temperature on the quality of horned galls, particularly cultivated ones.

## Discussion

### Development of SSR markers of *R. chinensis* and *S. chinensis*

SSR markers have been widely developed in plants for genetic diversity analysis, parentage assessment, species identification, and genetic map constructions [31–35]. Since no reference genome is available for *R. chinensis*, the transcriptome and reduced-representation genomes were used to develop SSRs in this study. The SSRs derived via these two approaches consistently revealed high polymorphism but no significant difference (See Supplementary Table 4, Additional File 1). Generally, genomic SSRs show variations in genetic information, while transcriptomic SSRs are associated with phenotypic traits [36, 37]. An integrated analysis using both kinds of SSRs in the present study reflected the relatively real level of genetic diversity in the *R. chinensis* trees.

Nine pairs of high-quality genomic SSR markers were developed for *S. chinensis* (See Supplementary Table 4, Additional File 1). The previous genetic analysis of *S. chinensis* was based on the SSR markers of the closely related species, while the amplified loci were extremely limited with a mean value of 3.00 alleles per locus [38]. We mined SSR motifs from the whole genome of *S. chinensis* and converted them to available markers with an outstanding polymorphism. And the alleles number had a qualitative improvement with the mean value of 9.44 alleles per locus.

### Genetic analysis of host trees and aphids

The adaptation and strict dependence of parasites on their host plants would lead to strong genetic variation in parasites [39]. In many plant–insect interaction systems, such as the *Pinus*-*Arceuthobium americanum* system, parasites evidenced higher genetic diversity than their host plants [40]. Consistent with these earlier reports, our study demonstrated that the level of genetic diversity in aphids was higher than that in its host, *R. chinensis* (See Supplementary Fig. 5, Additional File 1). The dual selection pressures from the environment and their hosts on the horned gall parasites possibly contributed to the increased genetic variation.

Generally, the genetic diversity of crop cultivars is lower than that of the wild individuals or their closely related relatives. The genome resequencing of wild and cultivated soybean suggested the loss of approximately half of the genetic diversity during the domestication of wild individuals into cultivars [41]. Similarly, the whole-genome sequencing of cultivated and wild peppers demonstrated higher diversity in the wild accessions than cultivars [42]. Studies on various crop species such as rice [43], cucumber [44], maize [45], watermelon [46], and potato [47] further confirmed that the nucleotide diversity of wild resources was higher than cultivars. The WJP populations of our study followed this general rule with the wild individuals having higher genetic diversity than the cultivars for both plants and aphids. These observations suggest a loss of genetic diversity in cultivated samples during the prolonged artificial selection and domestication [41, 42].

However, the level of genetic diversity was not significantly different between the wild and cultivated samples from other locations (See Supplementary Fig. 5, Additional File 1). Although the phenomenon was paradoxical to the results of WJP, it can be explained well when considering germplasm’s original and cultivation history. Comparatively, WJP has been the center of selection and domestication of both *R. chinensis* trees and *S. chinensis* aphids for several decades, which induced selective pressure on the cultivated samples. Consequently, both trees and aphids substantially lost genetic diversity during the breeding process (See Supplementary Fig. 5, Additional File 1). However, the other locations, such as BNG, BLZ, HLP, and HS, are the production bases of horned galls developed recently by collecting host plants and aphid germplasms all over Wufeng County. Thus, the levels of genetic diversity in these four populations were still close to those of wild germplasm although the differentiation was beginning to present between them (See Supplementary Fig. 5, Additional File 1).

### Both genetic and ecological factors associated with the quality degradation of horned galls

The quality of the horned galls, particularly the cultivated ones from Wufeng, showed a recent decline. Among the various samples, the WJP gall samples showed the highest degradation based on gallic acid content (Fig. 1A and B). Studies have confirmed that multiple factors, such as genotypes, environments, and their interactions significantly influence plant growth [14, 22, 30]. Our results showed that genetic and climatic factors potentially contributed to the quality degradation of horned galls. For example, the host trees from WJP and BLZ showed clear genetic differentiation (Fig. 4C, Supplementary Figs. 6C and D, Additional File 1). Consistent with these differences, the climatic factors, particularly the annual average and growing-season average temperatures, significantly varied between WJP and BLZ (Fig. 5A and B). These observations suggest that the annual average temperature and the growing-season average temperature might have contributed to the quality differentiation between WJP and BLZ horned galls.

Temperature, sunshine, precipitation, and relative humidity are the major environmental factors affecting plant growth characteristics [20, 48, 49]. Our study compared eight ecological factors and found differences in temperature indicators among the five locations. These temperature differences combined with the genetic differentiations explained the quality degradation in WJP (Fig. 5). Previous correlation analysis between the annual mean temperature, the annual mean sunshine hours, the annual mean precipitation, and the annual mean relative humidity with the gallic acid content demonstrated that only temperature was significant negatively correlated with the quality of horned galls [38]. Our present study confirmed the negative influence of temperature on horned galls. However, the correlation coefficient revealed only a moderate effect of temperature (0.5 <|r|< 0.8) on gallic acid content, which reinforced that temperature was a secondary factor affecting the quality of horned galls [38]. Consistent with this argument, although the temperature of BLZ was significantly different from that of the other four locations, the quality of the horned galls only appeared to be different among BLZ and WJP. Thus, these observations and earlier reports suggest that the impact of temperature was secondary as a single factor cannot lead to significant quality differentiation in horned galls.

Numerous studies have revealed that the content of active ingredients in plants were comprehensively adjusted by a combination of genetic and environmental factors, with the genetic factors having a major impact. The accumulation of pharmacologically active ingredients of *Eucommia ulmoides* was affected by genotype, environmental factors, and their interaction, with genotype acting as the main influencing factor [18]. The genetic factor was the most significant one affecting the metabolite profiles and concentrations in brassica vegetables, while the environmental and agronomic factors were secondary [50]. The present study found that prolonged cultivation and domestication of the host plant, *R. chinensis*, led to a partial loss of genetic diversity. The selectively retained individuals were mostly characterized by high yields but not good quality. As a result, the genetic richness of the cultivated population was reduced under long-term domestication, which further contributed to a decline in the quality of horned galls. Multiple studies have shown that Galla chinensis is suitable for growth in relatively cold environments, such as the BLZ in our research. Therefore, in addition to the influence of genetic factors, an appropriate temperature is crucial for promoting the growth and development of Galla chinensis and the accumulation of active ingredients. Thus, our findings suggest that both genetic and ecological factors (especially the temperature here) lead to the quality degradation of horned galls, particularly the cultivated ones, among which the former was the primary reason.

Generally, there should be a trade-off between the presence of metabolic and morphological traits of plants under stably growing conditions. Different to the metabolic trait of gallic acid content, both morphological traits of fresh weight and gall size were more easily subjected to artificial selection during cultivation, leading to high yields of cultivated horned galls but low genetic diversity as evidenced in the present study (See Supplementary Figs. 2A, B, C, and Supplementary Table 1, Additional File 1). Moreover, a positive correlation (*r* = 0.86, *P* < 0.01) was observed between the fresh weight and gall size of horned galls (See Supplementary Table 2, Additional File 1). Furthermore, the microenvironment (such as soil and light) of the wild populations was more complex and diverse than that of the cultivated populations. The differences in microhabitat conditions might have significant contributed to the differences in medicinal components and morphological traits among wild individuals. Thus, we suggest that the genetic background of the host plant is vital for the quality of horned galls. Besides, other factors, such as the microhabitat, also influence the quality of Galla chinensis.

Thus, given the genetic differentiation and quality degradation of horned galls under the continuous and directional selection for high yield, collecting diverse germplasm from areas outside Wufeng County and reinforcing the core collection for breeding is necessary. Moreover, selecting good germplasm of both *R. chinensis* and *S. chinensis* is important for horned galls production in terms of a good balance between metabolic and morphological qualities.

## Conclusions

Long-term monoculture led to the quality degradation of horned galls, primarily influenced by internal genetic and external ecological factors. Continuous artificial cultivation specifically led to genetic differentiation in the *R. chinensis* trees, degrading the quality of horned galls. Therefore, high polymorphic SSR markers were developed to assist in collecting and protecting the wild germplasm of both *R. chinensis* and *S. chinensis.* These findings will help to maintain the genetic basis of the cultivated germplasm and improve the production and medical and industrial applications of horned galls.

## Methods

### Sample collection, DNA extraction, and phenotypic trait measurement

Galla chinensis was initially identified by Dr. Ziyang Sang of the Forestry Science Research Institute of Wufeng County. The voucher specimen of Galla chinensis has been stored at the herbarium of traditional Chinese medicine of Hubei University of Chinese Medicine (voucher number: 202210GCWF). The compound leaves bearing horned galls of 102 cultivated and wild samples were collected in October 2021 from WJP, HLP, BNG, BLZ, and HS locations of Wufeng County, Hubei Province, China (See Supplementary Table 6, Additional File 1). The horned galls that grew with other woody and herbaceous plants in mountain forests with less human disturbance were collected as wild samples. These wild galls were the product of the natural migration of parasitic aphids with no human manipulation of hanging aphids. Meanwhile, the horned galls that grew in the artificial cultivation fields, with manually hung parasitic aphids, were collected as the cultivated samples. And these fields were under regular maintenance such as weeding and tree pruning.

The wild and cultivated horned galls from five villages across Wufeng County were used in this study. The sampling was carried out as follows: each population was randomly sampled, maintaining at least 15 m between the individuals. The galls and leaves from each sample were separated. The leaves were preserved in silica gel for genomic DNA extraction. Meanwhile, a gall was randomly selected, cut to expose the inner aphids and stored in absolute ethanol at -20 ℃ for further genomic DNA extraction from the parasites. The remaining galls were retained to measure the phenotypic traits. As the distance between the wild and cultivated populations in a single location was within 10 km, the climatic conditions are considered to be the same. Data on the climatic and environmental factors of each location are shown in Fig. 5.

The leaves of the host plants were ground into powder for genomic DNA extraction by a modified cetyltrimethy-lammonium bromide (CTAB) method [51]. The aphids were immersed in distilled water for 24 h and then were broken up using a tissue grinder in 1.5 mL Eppendorf tubes before the DNA extraction employing phenol/chloroform method [29].

Three galls were randomly selected from a single host tree to evaluate the phenotypic traits, such as the gall fresh weight, gall size, and wall thickness. Due to the irregular shape of horned galls, the size of galls was converted to the volume of water using the displacement method. The wall thickness was measured at three different regions of a gall and represented as a mean of these values.

### High performance liquid chromatography (HPLC) analysis of gallic acid

The sampled horned galls were first boiled for two minutes, dried for seven hours under 100 ℃, and cracked to remove the aphids and other impurities inside for analysing the gallic acid content [52]. The dried galls were ground into powder and passed through a 65-mesh sieve. 0.5 g of this powder was transferred to a conical flask containing 50 mL of 4 M hydrochloric acid and heated in a 95 ℃ water bath for 3.5 h to extract the gallic acid. After cooling and filtering the extraction solvent, 1 mL of the filtrate was added to a 100 mL volumetric flask and diluted with 50% methanol (v/v) to graduation. The final extract was shaken and filtered through a 0.45 µm PTFE filter. The reference standard of gallic acid (CAS: 149-91-7) was purchased from Shanghai Yuanye Bio-Technology Co., Ltd. (Shanghai, China) and was dissolved in 50% methanol to obtain the final standard solution at a concentration of 40 µg mL^−1^.

HPLC analysis was performed on a Shimadzu LC-20AD HPLC system equipped with an SPD-20A detector and CTO-20A thermostatic column compartment (Shimadzu, Kyoto, Japan) using an Ultimate XB-C18 column (250 mm × 4.6 mm, 5 µm; Shanghai Welch Technology Co., Ltd., Shanghai, China). The binary elution system used methanol and 0.1% phosphoric acid as solvents A and B. The elution was carried out using 5% A and 95% B for 15 min. The column temperature was set to 40 ℃ at a flow rate of 1.0 mL min^−1^. Then, 20 μL of the samples was loaded into the system, and the gallic acid was detected with a UV–VIS detector at 273 nm (See Supplementary Fig. 8, Additional File 1). A standard curve established using the concentration of gallic acid standard as the abscissa (*x*) and the peak area as the ordinate (*y*) showed a good linear relationship (*R*^2^ = 0.9993) (See Supplementary Table 7, Additional File 1).

### RNA-sequencing and double digest restriction association DNA (dd-RAD) sequencing

Total RNA was extracted from the compound leaves of *R. chinensis* using TRIzol reagent (Thermo Fisher Scientific, Waltham, MA, United States) according to the manufacturer’s instructions. Then, cDNA libraries were prepared using the extracted RNA and sequenced on the Illumina NovaSeq 6000 sequencing platform (Illumina, Inc., San Diego, CA, United States). The raw sequencing reads were filtered to obtain the high-quality RNA-seq data, and transcriptome assembly was accomplished using Trinity software [53]. The functions of the assembled unigenes were annotated based on the NCBI non-redundant protein sequences (Nr, http://www.ncbi.nlm.nih.gov), Kyoto Encyclopedia of Genes and Genomes Ortholog (KEGG, https://www.genome.jp/kegg/) [54–56], Clusters of Orthologous Groups of proteins (COG, http://www.ncbi.nlm.nih.gov/COG), Gene Ontology (GO, http://geneontology.org/), Unified Protein (UniProt, https://www.uniprot.org/), and the protein families (Pfam, http://pfam-legacy.xfam.org/) databases.

For ddRAD-seq, 100 ng of the genomic DNA was double digested with 5 U of *Sac* I and *Mse* I (New England Biolabs (Beijing) Ltd., Beijing, China) in a 25 µL reaction containing 1 × restriction buffer. The enzyme product was ligated with the restriction fragment utilizing the SacAD and MseAD adaptors. Then the quantitative ligated products were pooled and purified, and the DNA fragments were further enriched by PCR with the KOD-Plus-Neo polymerase (TOYOBO (Shanghai) Biotech Co. Ltd., Shanghai, China). Fragments varied from 500 to 550 bp were selected for final library construction with the Agilent DNA 12,000 kit using the 2100 Bioanalyzer system (Agilent Technologies (China) Co., Ltd., Beijing, China). The libraries were sequenced on the Illumina NovaSeq 6000 platform following the paired-end 150 (PE150) strategy.

### SSR marker development and amplification

Simple sequence repeats (SSRs) were searched on the sequences generated by RNA sequencing and dd-RAD sequencing of *R. chinensis* and the genome of *S. chinensis* (Accession: GCA_019022885.1) [57] using a MicroSAtellite identification tool (MISA) version 2.1 with the default parameters [58]. The SSR primers were designed using Primer 3 software [59]. The forward primers were added with an M13 tail sequence (GTAAAACGACGGCCAGT) labelled with FAM (blue), HEX (green), and ROX (red).

The genomic DNA was then amplified in a 10 μL reaction system containing 2 μL genomic DNA, 5 μL 2 × Taq PCR MasterMix, 0.04 μL forward primer, 0.25 μL reverse primer, 0.15 μL M13-FAM/M13-HEX/M13-ROX, and 2.56 μL ultrapure water. The mixed PCR amplifications were performed on a BiometraTone 96G (Analytik Jena AG, Jena, Germany) and the PCR products were detected by automatic fluorescence using an ABI 3730XL Sequence Analyzer for primer screening. The SSR primers with high amplification efficiency, good reproducibility, and high polymorphism were retained for the overall genetic analysis. GeneMarker® software was used to analyze the amplified fragment size of the different samples at each SSR locus.

### Ecological factor extraction and analysis

The latitude and longitude of the five locations were imported into Wheat A (version 1.4.9) to extract the monthly data on ecological factors for the past decade (2012 to 2021) with an accuracy of 10 kms. The key ecological factors, including mean annual temperature (MAT), mean growing-season (May to October) temperature (MGST), mean annual sunshine hours (MASH), mean growing-season sunshine hours (MGSSH), mean annual precipitation (MAP), mean growing-season precipitation (MGSP), mean annual relative humidity (MARH), and mean growing-season relative humidity (MGSRH), were fetched for the latter statistic. Data on all ecological factors used in this study were derived from The Famine Early Warning Systems Network Land Data Assimilation System (FLDAS), provided by the National Aeronautics and Space Administration (NASA) and the Famine Early Warning Systems Network (FEWS NET) with 0.1° × 0.1° spatial resolution and monthly temporal resolution [60].

### Statistical analysis

The ordinary one-way ANOVA analyses of the gallic acid content and ecological factors among populations were conducted using GraphPad Prism 8.3.0 for windows (GraphPad Software, San Diego, California USA, www.graphpad.com.). The Principal Component Analysis (PCA) analysis was performed using SIMCA® 17. The genetic diversity parameters such as allele frequency, number of alleles (*N*_A_), effective number of alleles (*N*_E_), observed heterozygosity (*H*_O_), expected heterozygosity (*H*_E_), Shannon’s information index (*I*), Nei’s gene diversity index (*H*), fixation index (*F*_ST_), and gene flow (*N*_*m*_) were calculated using the POPGENE software (version 1.3.2) [61]. PIC Calc software [62] was used to estimate the polymorphism information content (*PIC*). Neighbour-Joining (NJ) cluster analysis was carried out using the Analysis of Phylogenetics and Evolution (APE) package [63] in R (R Core Team, 2022), and the NJ tree was visualized utilizing the iTOL online tool (version 6.5.8) [64]. GenAlEx (version 6.5) [65] was used to perform the analysis of molecular variance (AMOVA). The population structure was determined by STRUCTURE software (version 2.0) [66] and the number of genetic groups was detected using the STRUCTURE HARVESTER web-based program [67].

### Supplementary Information

**Additional file 1:** **Supplementary Figure 1.** Different types of Galla Chinensis. (A) Horned gall. (B) Gallnut. (C) Flower-like gall. **Supplementary Figure 2.** Analysis of the key phenotypic traits. Comparative analysis of gallic acid (A), fresh weight (B), gall size (C), and wall thickness (D) between wild and cultivated horned galls. The fresh weight (E), gall size (G), wall thickness (I) of the horned galls from five locations. And the fresh weight (F), gall size (H), wall thickness (J) of wild and cultivated horned galls in each location. (*, *P* < 0.05; **, *P* < 0.01; ***, *P* < 0.001; ****, *P* < 0.0001). **Supplementary Figure 3.** Principal Component Analysis (PCA) analysis of Bai Nianguan (BNG) (A), Bai Luzhuang (BLZ) (B), Huang Liangping (HLP) (C), and Huo Shan (HS) (D) populations based on phenotypic traits. **Supplementary Figure 4.** Function annotation of *Rhus chinensis* unigenes on the basis of public database. (A) Summary of annotations of unigenes in six databases. (B) GO classification of annotated unigenes. (C) Functional classification of unigenes based on the KEGG pathway. (D) The COG functional distribution of annotated unigenes. **Supplementary Figure 5.** The genetic paraments of each *R. chinensis* and *Schlechtendalia chinensis* population (WJP, BNG, BLZ, HLP, and HS represent the population from Wang Jiaping, Bai Nianguan, Bai Luzhuang, Huang Liangping, and Huo Shan, respectively.). (A). The number of alleles (*N*). (B) The average number of alleles (*N*_A_). (C) The effective number of alleles (*N*_E_). (D) observed heterozygosity (*H*_O_). (E) expected heterozygosity (*H*_E_). (F) Shannon’s information index (*I*). (G) Nei’s gene diversity index (*H*). (H) Polymorphism information content (*PIC*). **Supplementary Figure 6.** STRUCTURE and PCA analysis of host trees and aphids based on SSR (Simple Sequence Repeat) loci. (A) Population structure of 102 *R. chinensis* accessions. (B) Population structure of 102 *S. chinensis* accessions. (C) Population structure of *R. chinensis* accessions in WJP and BLZ. (D) PCA analysis of *R. chinensis* accessions in WJP and BLZ. (E) Population structure of *R. chinensis* accessions in WJP. (F) PCA analysis of *R. chinensis* accessions in WJP. **Supplementary Figure 7.** Neighbour-Joining (NJ) analysis based on the SSR loci information of *R. chinensis *accessions. NJ tree of individuals from BNG (A), BLZ (B), HLP (C), and HS (D). The red stars represent the wild accessions and the blue stars represent the cultivated accessions. **Supplementary Figure 8. **Chromatograms of negative sample (A), gallic acid standard (B), and horned gall sample (C). **Supplementary Table 1.** Phenotypic data among 102 horned galls. **Supplementary Table 2.** Correlation analysis of phenotypic traits. **Supplementary Table 3.** Statistic of RNA-seq, ddRAD-seq data to *R. chinensis *and the reference genome data of *S. chinensis*. **Supplementary Table 4.** Genetic characterization of 23 pairs of *R. chinensis* SSR primers and 9 pairs of *S. chinensis* SSR primers.** Supplementary Table 5.** Analysis of molecular variance (AMOVA) for *R. chinensis* and *S. chinensis* populations. **Supplementary Table 6.** Sampling information. **Supplementary Table 7.** Method validation of the high-performance liquid chromatography (HPLC) analysis in horned galls.

## Data Availability

The raw sequence data generated during the current study are available in the Genome Sequence Archive (Genomics, Proteomics & Bioinformatics 2021) in National Genomics Data Center (Nucleic Acid Res 2022), China National Center for Bioinformation /Beijing Institute of Genomics, Chinese Academy of Science (GSA: CRA008302) that are publicly accessible at https://ngdc.cncb.ac.cn/gsa/s/Qfnrun09.

## Abbreviations

TCM: Traditional Chinese Medicine
WJP: Wang Jiaping
BNG: Bai Nianguan
BLZ: Bai Luzhuang
HLP: Huang Liangping
HS: Huo Shan
PCA: Principal Component Analysis
SSR: Simple Sequence Repeat
*Rc*-tSSR: Transcriptomic SSRs of *R. chinensis*
*Rc*-gSSR: Genomic SSRs of *R. chinensis*
*Sc*-gSSR: Genomic SSRs of *S. chinensis*
RNA-seq: RNA sequencing
dd-RAD: Double digest Restriction Association DNA
*N*_A_: Number of alleles
*N*_E_: Effective number of alleles
*H*_O_: Observed heterozygosity
*H*_E_: Expected heterozygosity
*I*: Shannon’s information index
*H*: Nei’s gene diversity index
*F*_ST_: Fixation index
*N*_*m*_: Gene flow
*PIC*: Polymorphism Information Content
NJ tree: Neighbour-Joining tree
APE: Analysis of Phylogenetics and Evolution
AMOVA: Analysis of molecular variance
CTAB: Cetyltrimethy-lammonium bromide
HPLC: High performance liquid chromatography
Nr: NCBI non-redundant protein sequences database
KEGG: Kyoto Encyclopedia of Genes and Genomes Ortholog database
COG: Clusters of Orthologous Groups of proteins
GO: Gene Ontology
UniProt: Unified Protein
Pfam: Protein families database
MAT: Mean annual temperature
MGST: Mean growing-season (May to October) temperature
MASH: Mean annual sunshine hours
MGSSH: Mean growing-season sunshine hours
MAP: Mean annual precipitation
MGSP: Mean growing-season precipitation
MARH: Mean annual relative humidity
MGSRH: Mean growing-season relative humidity
FLDAS: The Famine Early Warning Systems Network Land Data Assimilation System
NASA: National Aeronautics and Space Administration
FEWS NET: Famine Early Warning Systems Network
ANOVA: Analysis of Variance
