# Supplementary material for: Fine-Scale analysis of both wild and cultivated horned galls provides insight into their quality differentiation
Source: BMC Plant Biol. 2023 Sep 14;23:426. doi: 10.1186/s12870-023-04442-1 (PMC10500821; doi:10.1186/s12870-023-04442-1)
Supplement: Supplementary file 1 — Additional file 1: Supplementary Figure 1. Different types of Galla Chinensis. (A) Horned gall. (B) Gallnut. (C) Flower-like gall. Supplementary Figure 2. Analysis of the key phenotypic traits. Comparative analysis of gallic acid (A), fresh weight (B), gall size (C), and wall thickness (D) between wild and cultivated horned galls. The fresh weight (E), gall size (G), wall thickness (I) of the horned galls from five locations. And the fresh weight (F), gall size (H), wall thickness (J) of wild and cultivated horned galls in each location. (*, P < 0.05; **, P < 0.01; ***, P < 0.001; ****, P < 0.0001). Supplementary Figure 3. Principal Component Analysis (PCA) analysis of Bai Nianguan (BNG) (A), Bai Luzhuang (BLZ) (B), Huang Liangping (HLP) (C), and Huo Shan (HS) (D) populations based on phenotypic traits. Supplementary Figure 4. Function annotation of Rhus chinensis unigenes on the basis of public database. (A) Summary of annotations of unigenes in six databases. (B) GO classification of annotated unigenes. (C) Functional classification of unigenes based on the KEGG pathway. (D) The COG functional distribution of annotated unigenes. Supplementary Figure 5. The genetic paraments of each R. chinensis and Schlechtendalia chinensis population (WJP, BNG, BLZ, HLP, and HS represent the population from Wang Jiaping, Bai Nianguan, Bai Luzhuang, Huang Liangping, and Huo Shan, respectively.). (A). The number of alleles (N). (B) The average number of alleles (NA). (C) The effective number of alleles (NE). (D) observed heterozygosity (HO). (E) expected heterozygosity (HE). (F) Shannon’s information index (I). (G) Nei’s gene diversity index (H). (H) Polymorphism information content (PIC). Supplementary Figure 6. STRUCTURE and PCA analysis of host trees and aphids based on SSR (Simple Sequence Repeat) loci. (A) Population structure of 102 R. chinensis accessions. (B) Population structure of 102 S. chinensis accessions. (C) Population structure of R. chinensis accessions in WJP and [file 12870_2023_4442_MOESM1_ESM.doc]

**Additional File 1**


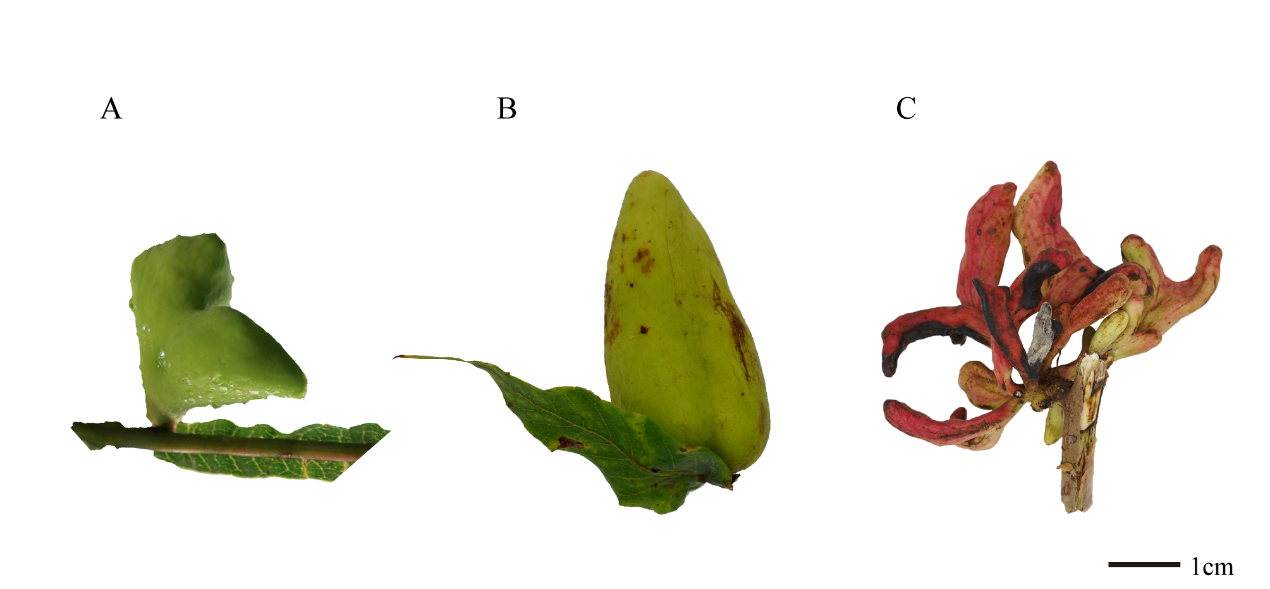


**Supplementary Figure 1.** Different types of Galla Chinensis. (A) Horned gall. (B) Gallnut. (C) Flower-like gall.


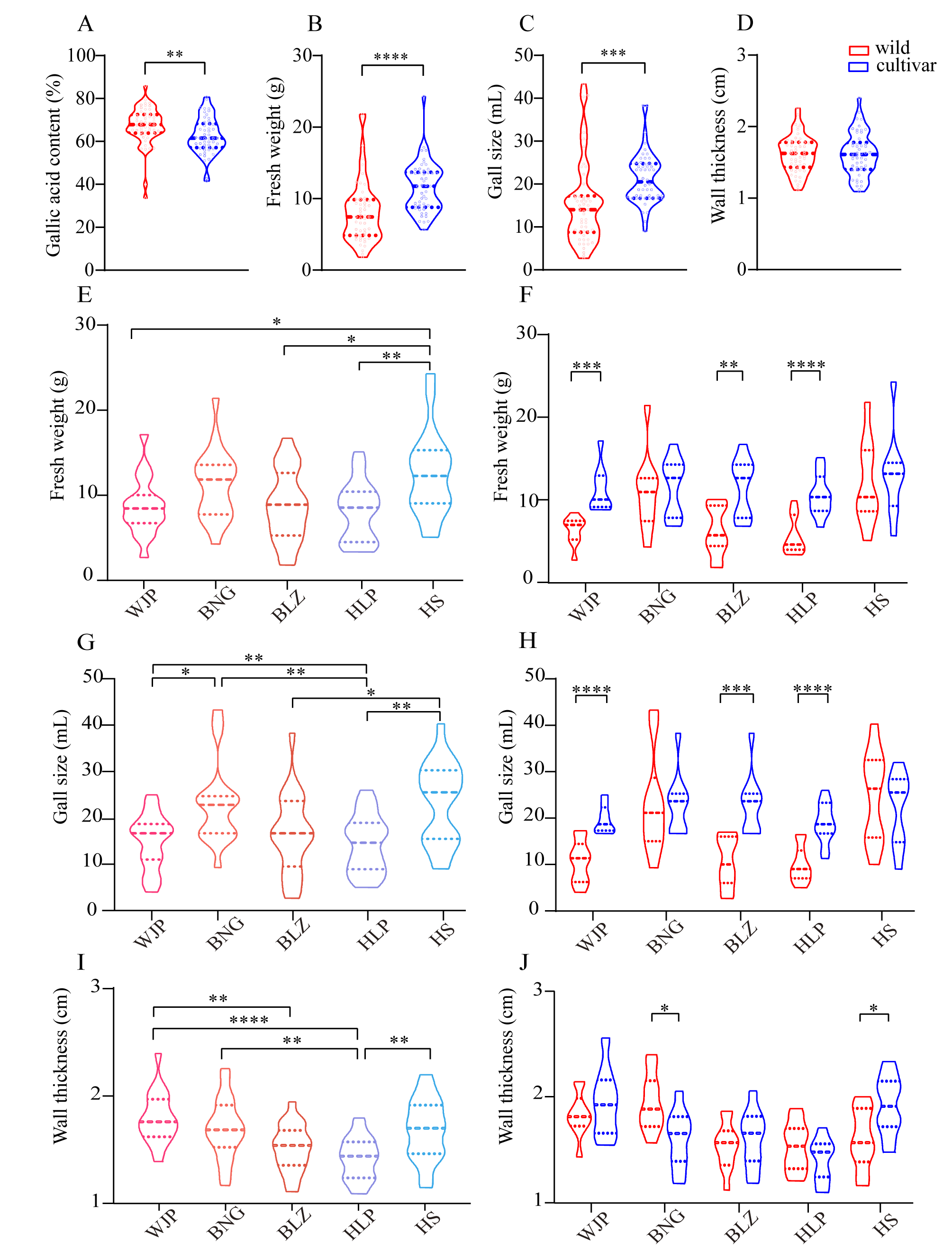


**Supplementary Figure 2.** Analysis of the key phenotypic traits. Comparative analysis of gallic acid (A), fresh weight (B), gall size (C), and wall thickness (D) between wild and cultivated horned galls. The fresh weight (E), gall size (G), wall thickness (I) of the horned galls from five locations. And the fresh weight (F), gall size (H), wall thickness (J) of wild and cultivated horned galls in each location. (*, P < 0.05; **, P < 0.01; ***, P < 0.001; ****, P < 0.0001)


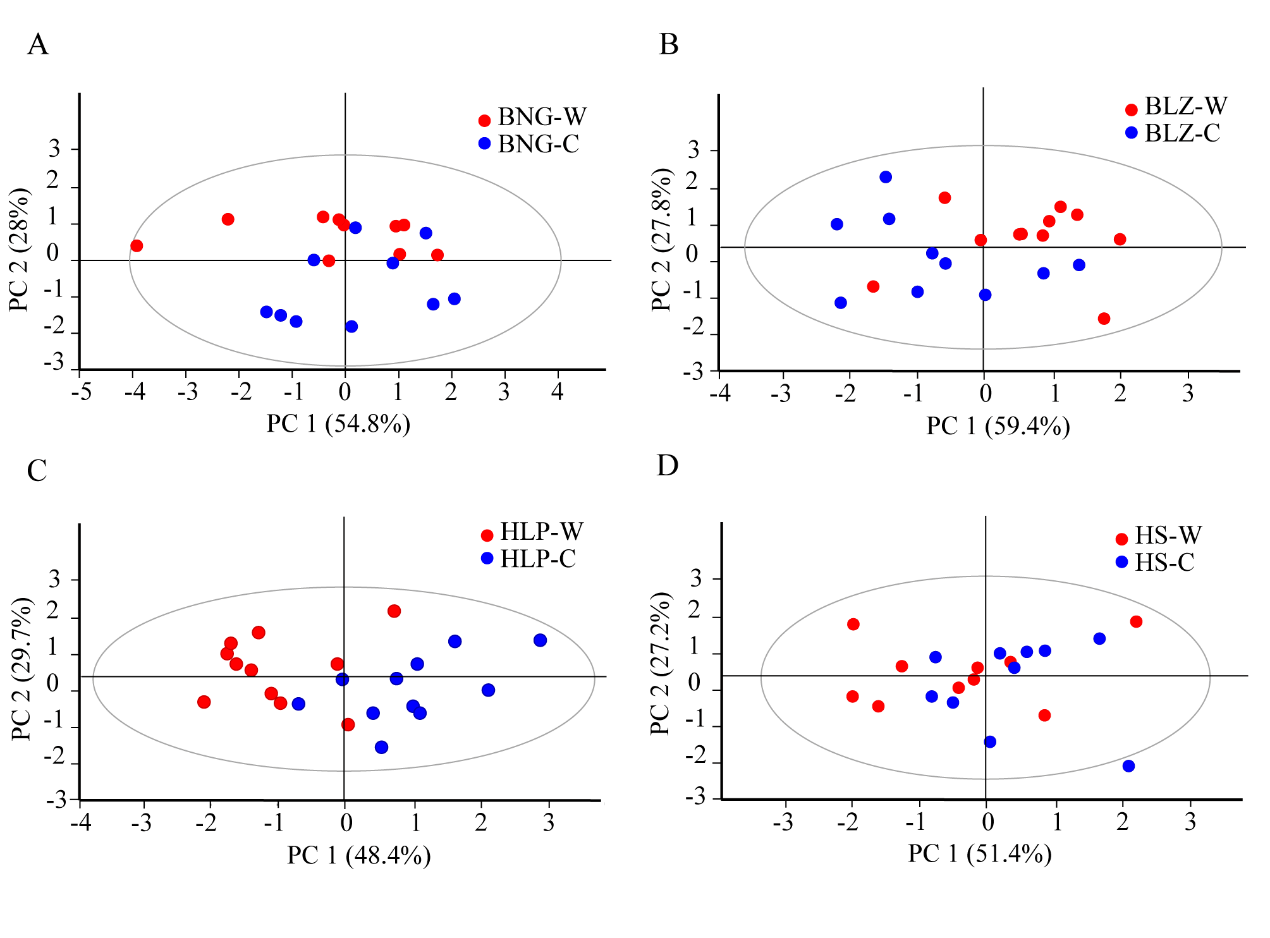


**Supplementary Figure 3.** Principal Component Analysis (PCA) analysis of Bai Nianguan (BNG) (A), Bai Luzhuang (BLZ) (B), Huang Liangping (HLP) (C), and Huo Shan (HS) (D) populations based on phenotypic traits.


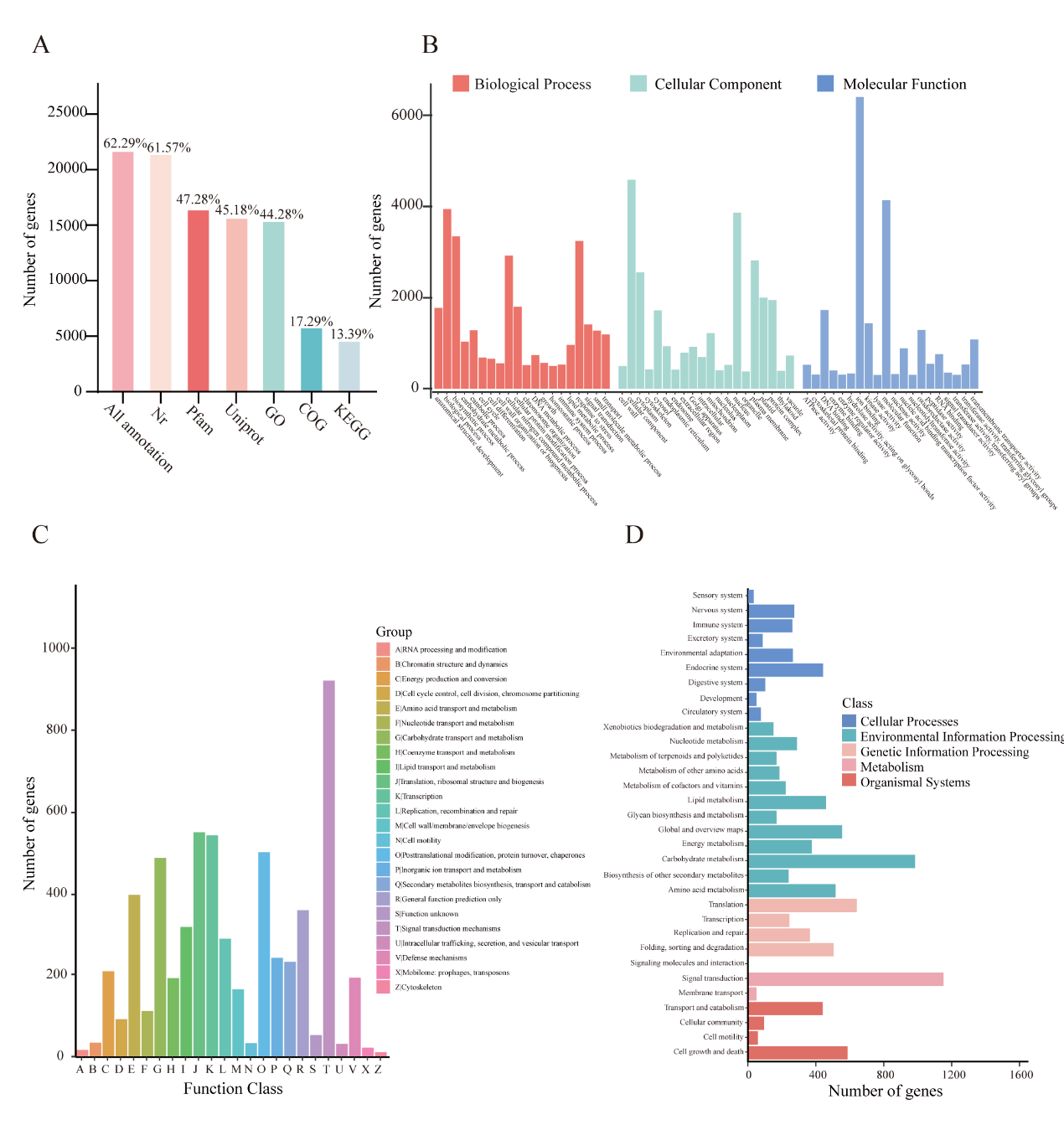


**Supplementary Figure 4.** Function annotation of *Rhus chinensis* unigenes on the basis of public database. (A) Summary of annotations of unigenes in six databases. (B) GO classification of annotated unigenes. (C) Functional classification of unigenes based on the KEGG pathway. (D) The COG functional distribution of annotated unigenes.


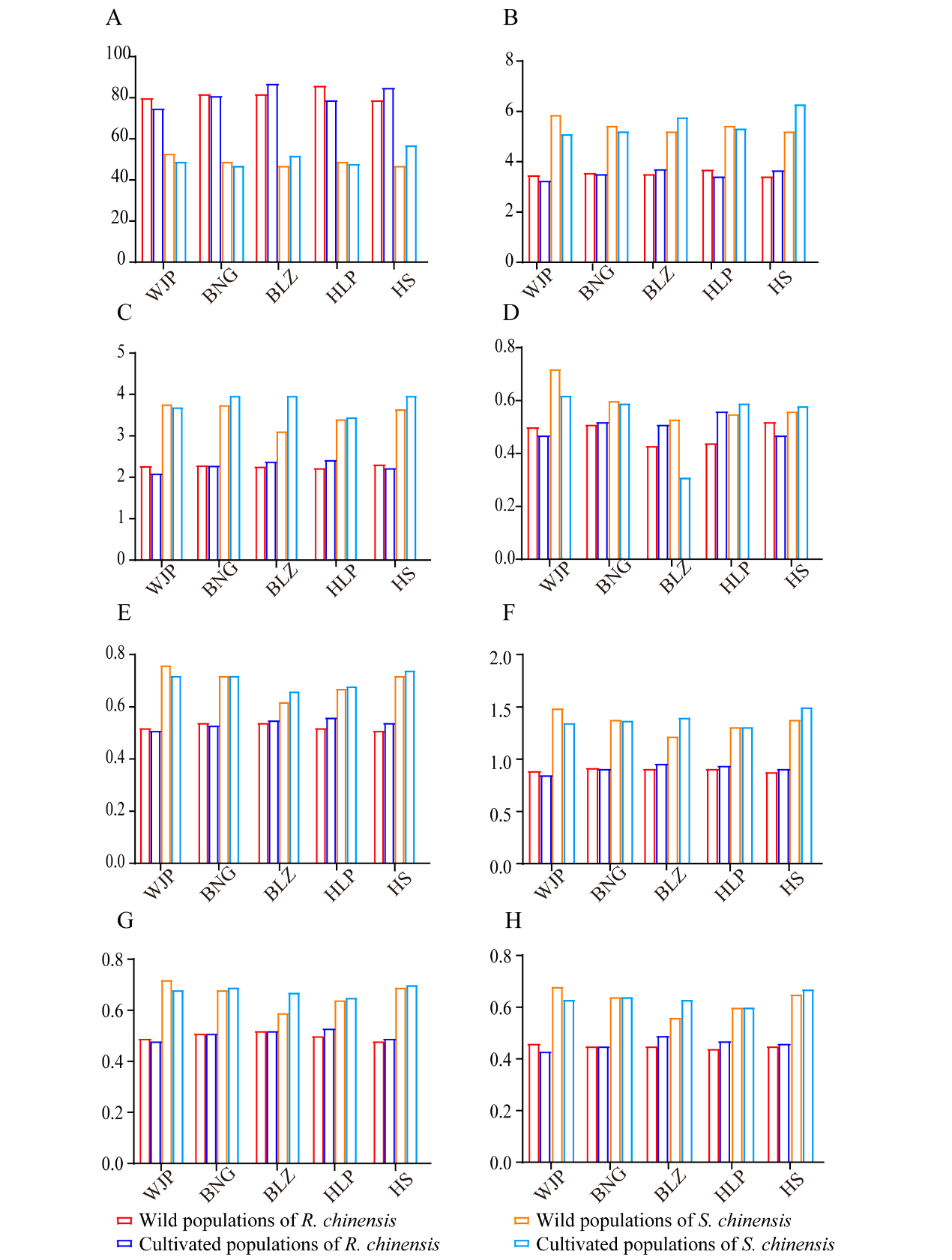


**Supplementary Figure 5.** The genetic paraments of each *R. chinensis* and *Schlechtendalia chinensis* population (WJP, BNG, BLZ, HLP, and HS represent the population from Wang Jiaping, Bai Nianguan, Bai Luzhuang, Huang Liangping, and Huo Shan, respectively.). (A). The number of alleles (*N*). (B) The average number of alleles (*N*_A_). (C) The effective number of alleles (*N*_E_). (D) observed heterozygosity (*H*_O_). (E) expected heterozygosity (*H*_E_). (F) Shannon’s information index (*I*). (G) Nei’s gene diversity index (*H*). (H) Polymorphism information content (*PIC*).


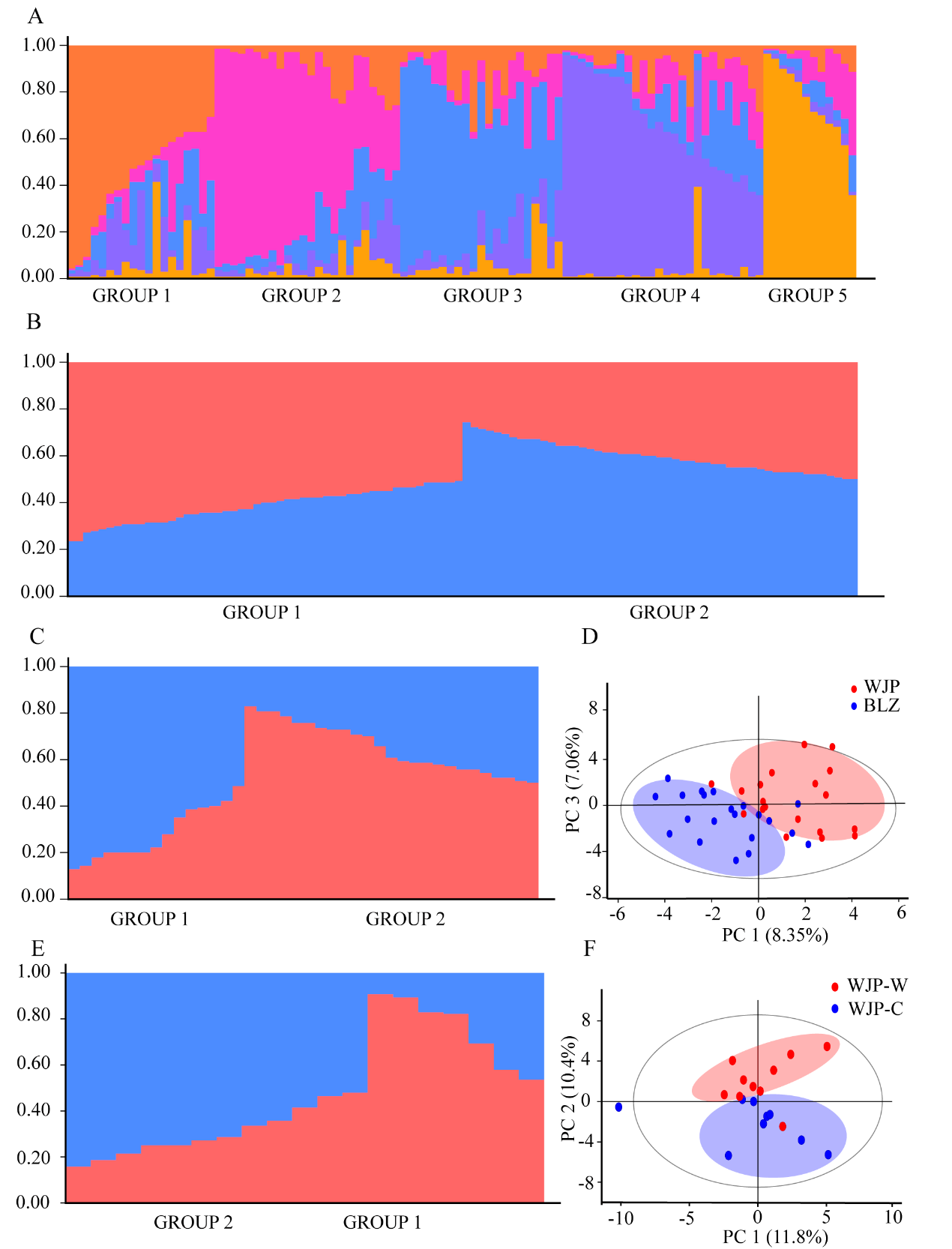


**Supplementary Figure 6.** STRUCTURE and PCA analysis of host trees and aphids based on SSR (Simple Sequence Repeat) loci. (A) Population structure of 102 *R. chinensis* accessions. (B) Population structure of 102 *S. chinensis* accessions. (C) Population structure of *R. chinensis* accessions in WJP and BLZ. (D) PCA analysis of *R. chinensis* accessions in WJP and BLZ. (E) Population structure of *R. chinensis* accessions in WJP. (F) PCA analysis of *R. chinensis* accessions in WJP.


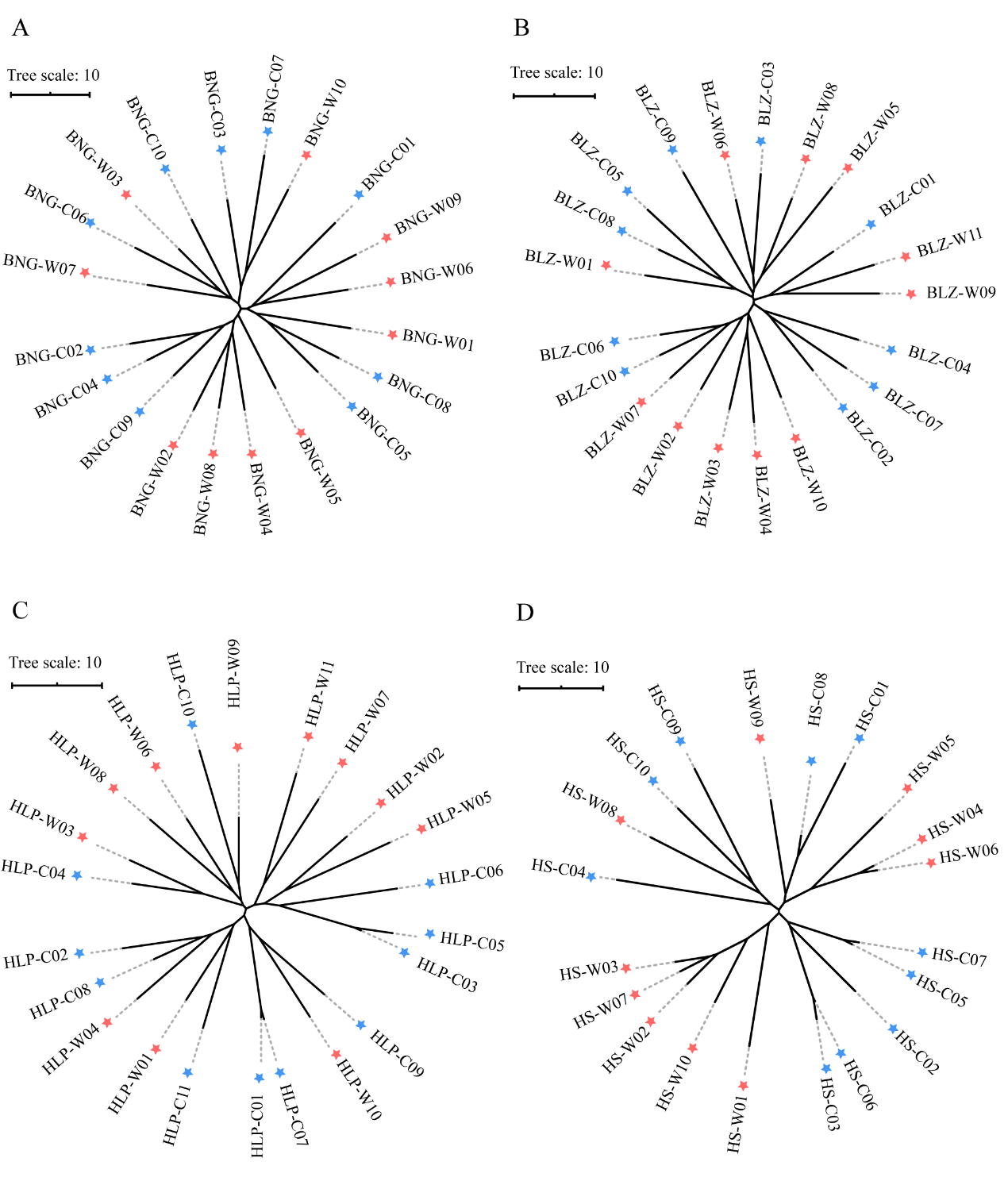


**Supplementary Figure 7.** Neighbour-Joining (NJ) analysis based on the SSR loci information of *R. chinensis* accessions. NJ tree of individuals from BNG (A), BLZ (B), HLP (C), and HS (D). The red stars represent the wild accessions and the blue stars represent the cultivated accessions.


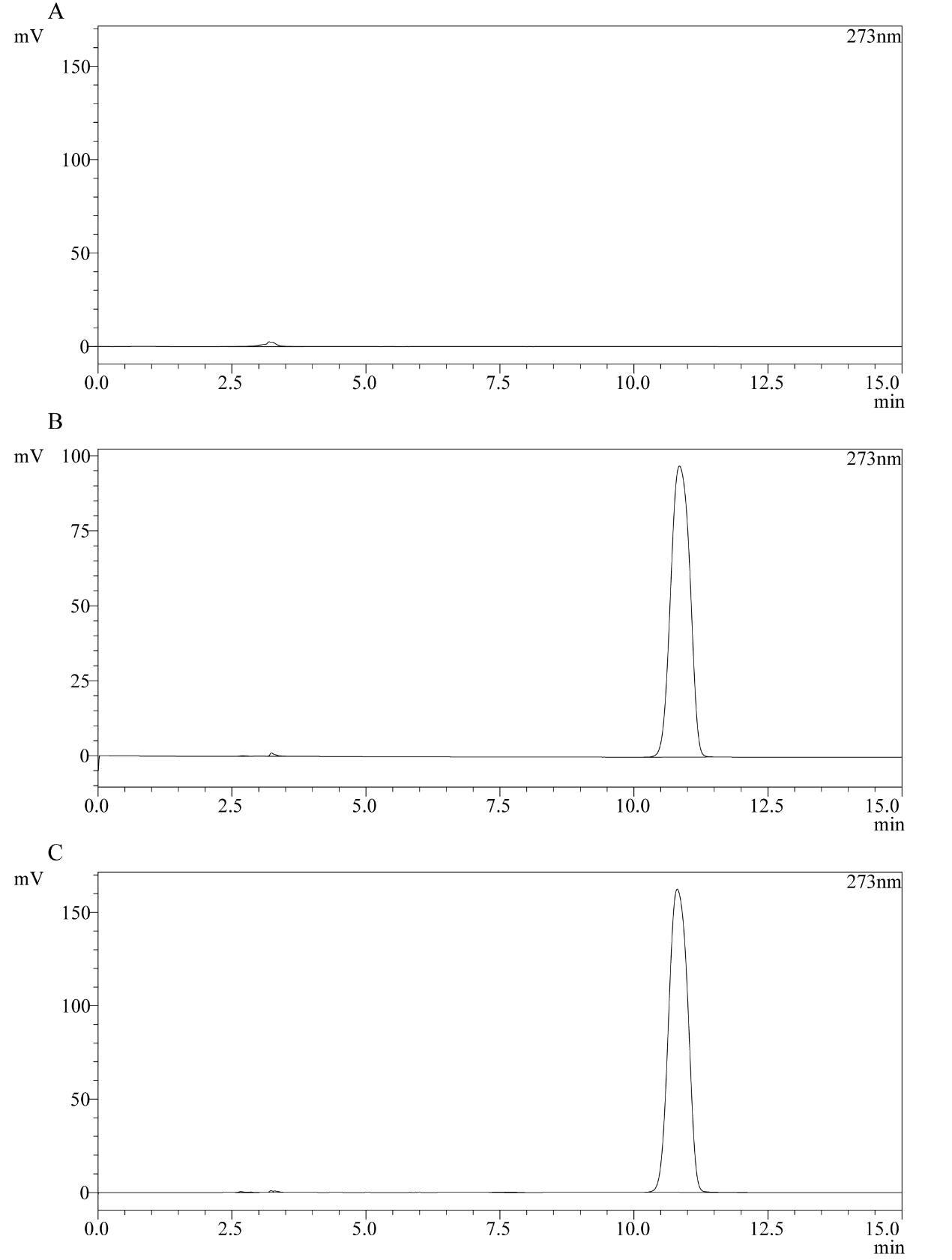


**Supplementary Figure 8.** Chromatograms of negative sample (A), gallic acid standard (B), and horned gall sample (C).

**Supplementary Table 1.** Phenotypic data among 102 horned galls.

| Population | Sample | Gallic acid content (%) | Fresh weight (g) | Gall size (mL) | Wall thickness (cm) |
| --- | --- | --- | --- | --- | --- |
| WJP-W | WJP-W-1 | 63.24 | 8.47 | 17.33 | 1.74 |
| WJP-W | WJP-W-2 | 65.25 | 6.73 | 11.67 | 1.39 |
| WJP-W | WJP-W-3 | 77.07 | 5.80 | 8.00 | 1.71 |
| WJP-W | WJP-W-4 | 67.81 | 7.20 | 13.67 | 1.66 |
| WJP-W | WJP-W-5 | 39.95 | 7.23 | 11.67 | 1.72 |
| WJP-W | WJP-W-6 | 65.30 | 7.60 | 11.00 | 1.77 |
| WJP-W | WJP-W-7 | 76.61 | 7.43 | 16.67 | 1.62 |
| WJP-W | WJP-W-8 | 65.33 | 5.27 | 6.33 | 1.84 |
| WJP-W | WJP-W-9 | 76.81 | 4.90 | 6.00 | 2.03 |
| WJP-W | WJP-W-10 | 61.73 | 2.70 | 4.00 | 2.02 |
| WJP-W | WJP-W-mean | 65.91 | 6.33 | 10.63 | 1.75 |
| WJP-C | WJP-C-1 | 56.52 | 9.25 | 25.00 | 1.76 |
| WJP-C | WJP-C-2 | 53.88 | 8.80 | 17.33 | 1.49 |
| WJP-C | WJP-C-3 | 42.78 | 9.37 | 16.67 | 1.83 |
| WJP-C | WJP-C-4 | 51.72 | 9.00 | 17.33 | 1.92 |
| WJP-C | WJP-C-5 | 58.97 | 17.17 | 23.33 | 2.40 |
| WJP-C | WJP-C-6 | 58.87 | 12.47 | 20.00 | 1.97 |
| WJP-C | WJP-C-7 | 56.91 | 11.87 | 18.67 | 1.58 |
| WJP-C | WJP-C-8 | 54.56 | 13.43 | 21.33 | 1.60 |
| WJP-C | WJP-C-9 | 53.31 | 10.03 | 17.33 | 2.11 |
| WJP-C | WJP-C-mean | 54.17 | 11.26 | 19.67 | 1.85 |
| BNG-W | BNG-W-1 | 71.16 | 7.70 | 15.00 | 1.78 |
| BNG-W | BNG-W-2 | 72.39 | 12.20 | 40.67 | 2.17 |
| BNG-W | BNG-W-3 | 65.28 | 6.57 | 9.33 | 1.64 |
| BNG-W | BNG-W-4 | 71.01 | 12.00 | 15.00 | 1.96 |
| BNG-W | BNG-W-5 | 74.96 | 9.33 | 16.67 | 1.51 |
| BNG-W | BNG-W-6 | 69.23 | 21.43 | 43.33 | 2.26 |
| BNG-W | BNG-W-7 | 72.59 | 9.88 | 24.67 | 1.99 |
| BNG-W | BNG-W-8 | 63.79 | 4.30 | 20.00 | 1.81 |
| BNG-W | BNG-W-9 | 67.63 | 13.50 | 24.00 | 1.65 |
| BNG-W | BNG-W-10 | 70.55 | 12.30 | 22.33 | 1.72 |
| BNG-W | BNG-W-mean | 69.86 | 10.92 | 23.10 | 1.85 |
| BNG-C | BNG-C-1 | 65.17 | 9.07 | 16.67 | 1.62 |
| BNG-C | BNG-C-2 | 73.76 | 7.90 | 16.67 | 1.40 |
| BNG-C | BNG-C-3 | 51.16 | 13.53 | 24.00 | 1.95 |
| BNG-C | BNG-C-4 | 56.01 | 16.73 | 27.00 | 1.75 |
| BNG-C | BNG-C-5 | 75.26 | 11.73 | 23.33 | 1.56 |
| BNG-C | BNG-C-6 | 59.67 | 7.50 | 18.67 | 1.25 |
| BNG-C | BNG-C-7 | 61.27 | 6.80 | 16.67 | 1.17 |
| BNG-C | BNG-C-8 | 67.32 | 14.10 | 24.67 | 1.73 |
| BNG-C | BNG-C-9 | 58.30 | 14.77 | 38.33 | 1.63 |
| BNG-C | BNG-C-10 | 55.93 | 13.60 | 24.00 | 1.39 |
| BNG-C | BNG-C-mean | 62.39 | 11.57 | 23.00 | 1.54 |
| BLZ-W | BLZ-W-1 | 75.73 | 1.80 | 2.67 | 1.32 |
| BLZ-W | BLZ-W-2 | 77.78 | 4.85 | 9.00 | 1.42 |
| BLZ-W | BLZ-W-3 | 79.48 | 9.30 | 17.00 | 1.30 |
| BLZ-W | BLZ-W-4 | 73.90 | 9.70 | 17.00 | 1.78 |
| BLZ-W | BLZ-W-5 | 71.44 | 4.45 | 7.00 | 1.61 |
| BLZ-W | BLZ-W-6 | 63.35 | 2.23 | 3.33 | 1.11 |
| BLZ-W | BLZ-W-7 | 56.67 | 10.05 | 16.00 | 1.77 |
| BLZ-W | BLZ-W-8 | 69.60 | 8.90 | 14.00 | 1.54 |
| BLZ-W | BLZ-W-9 | 72.96 | 5.70 | 10.00 | 1.46 |
| BLZ-W | BLZ-W-10 | 72.03 | 6.60 | 14.00 | 1.51 |
| BLZ-W | BLZ-W-11 | 74.95 | 4.40 | 6.00 | 1.54 |
| BLZ-W | BLZ-W-mean | 71.63 | 6.18 | 10.55 | 1.49 |
| BLZ-C | BLZ-C-1 | 58.25 | 9.07 | 16.67 | 1.62 |
| BLZ-C | BLZ-C-2 | 61.52 | 7.90 | 16.67 | 1.40 |
| BLZ-C | BLZ-C-3 | 64.80 | 13.53 | 24.00 | 1.95 |
| BLZ-C | BLZ-C-4 | 75.78 | 16.73 | 27.00 | 1.75 |
| BLZ-C | BLZ-C-5 | 65.72 | 11.73 | 23.33 | 1.56 |
| BLZ-C | BLZ-C-6 | 68.10 | 7.50 | 18.67 | 1.25 |
| BLZ-C | BLZ-C-7 | 71.27 | 6.80 | 16.67 | 1.17 |
| BLZ-C | BLZ-C-8 | 68.96 | 14.10 | 24.67 | 1.73 |
| BLZ-C | BLZ-C-9 | 53.97 | 14.77 | 38.33 | 1.63 |
| BLZ-C | BLZ-C-10 | 65.68 | 13.60 | 24.00 | 1.39 |
| BLZ-C | BLZ-C-mean | 65.41 | 11.57 | 23.00 | 1.54 |
| HLP-W | HLP-W-1 | 60.26 | 4.13 | 6.00 | 1.63 |
| HLP-W | HLP-W-2 | 61.92 | 4.60 | 9.00 | 1.80 |
| HLP-W | HLP-W-3 | 67.99 | 3.50 | 7.00 | 1.58 |
| HLP-W | HLP-W-4 | 77.88 | 9.90 | 16.00 | 1.73 |
| HLP-W | HLP-W-5 | 33.60 | 8.60 | 16.50 | 1.57 |
| HLP-W | HLP-W-6 | 65.18 | 8.20 | 13.00 | 1.48 |
| HLP-W | HLP-W-7 | 69.63 | 3.95 | 7.00 | 1.37 |
| HLP-W | HLP-W-8 | 56.85 | 5.67 | 11.00 | 1.29 |
| HLP-W | HLP-W-9 | 66.51 | 4.27 | 8.67 | 1.38 |
| HLP-W | HLP-W-10 | 66.27 | 5.50 | 9.33 | 1.19 |
| HLP-W | HLP-W-11 | 58.10 | 3.35 | 5.00 | 1.24 |
| HLP-W | HLP-W-mean | 62.20 | 5.61 | 9.86 | 1.48 |
| HLP-C | HLP-C-1 | 79.64 | 15.13 | 26.00 | 1.50 |
| HLP-C | HLP-C-2 | 57.23 | 8.50 | 23.33 | 1.32 |
| HLP-C | HLP-C-3 | 65.99 | 10.00 | 21.00 | 1.09 |
| HLP-C | HLP-C-4 | 63.07 | 8.73 | 17.33 | 1.12 |
| HLP-C | HLP-C-5 | 63.24 | 10.97 | 18.67 | 1.55 |
| HLP-C | HLP-C-6 | 61.76 | 10.33 | 17.33 | 1.45 |
| HLP-C | HLP-C-7 | 59.83 | 8.67 | 13.33 | 1.45 |
| HLP-C | HLP-C-8 | 59.06 | 13.90 | 23.33 | 1.43 |
| HLP-C | HLP-C-9 | 69.57 | 12.80 | 19.67 | 1.64 |
| HLP-C | HLP-C-10 | 41.55 | 10.77 | 16.67 | 1.22 |
| HLP-C | HLP-C-11 | 59.76 | 6.70 | 11.33 | 1.23 |
| HLP-C | HLP-C-mean | 61.88 | 10.59 | 18.91 | 1.36 |
| HS-W | HS-W-1 | 64.45 | 9.00 | 16.00 | 1.39 |
| HS-W | HS-W-2 | 71.56 | 10.90 | 30.50 | 1.83 |
| HS-W | HS-W-3 | 56.60 | 9.77 | 24.00 | 1.23 |
| HS-W | HS-W-4 | 64.20 | 9.20 | 16.00 | 1.79 |
| HS-W | HS-W-5 | 66.68 | 15.55 | 32.00 | 1.46 |
| HS-W | HS-W-6 | 68.78 | 14.73 | 28.67 | 1.47 |
| HS-W | HS-W-7 | 56.37 | 7.47 | 15.33 | 1.55 |
| HS-W | HS-W-8 | 85.90 | 21.83 | 40.33 | 1.67 |
| HS-W | HS-W-9 | 64.85 | 17.45 | 34.00 | 1.90 |
| HS-W | HS-W-10 | 68.52 | 5.10 | 10.00 | 1.15 |
| HS-W | HS-W-mean | 66.79 | 12.10 | 24.68 | 1.54 |
| HS-C | HS-C-1 | 61.57 | 14.15 | 28.00 | 1.54 |
| HS-C | HS-C-2 | 72.42 | 11.83 | 13.33 | 1.68 |
| HS-C | HS-C-3 | 57.16 | 12.75 | 29.50 | 2.01 |
| HS-C | HS-C-4 | 61.34 | 13.60 | 26.00 | 1.43 |
| HS-C | HS-C-5 | 75.36 | 8.43 | 15.33 | 2.10 |
| HS-C | HS-C-6 | 61.54 | 24.30 | 26.00 | 2.20 |
| HS-C | HS-C-7 | 70.79 | 9.55 | 17.00 | 1.96 |
| HS-C | HS-C-8 | 67.85 | 5.65 | 9.00 | 1.72 |
| HS-C | HS-C-9 | 80.71 | 15.50 | 32.00 | 1.92 |
| HS-C | HS-C-10 | 74.27 | 13.55 | 25.00 | 1.72 |
| HS-C | HS-C-mean | 68.30 | 12.93 | 22.12 | 1.83 |

**Supplementary Table 2.** Correlation analysis of phenotypic traits.

|  | Gallic acid content (%) | Fresh weight (g) | Gall size (mL) | Wall thickness (mm) |
| --- | --- | --- | --- | --- |
| Gallic acid content (%) | 1.00 | 0.05 | 0.05 | 0.04 |
| Fresh weight (g) | 0.05 | 1.00 | 0.86^**^ | 0.40^**^ |
| Gall size (mL) | 0.05 | 0.86^**^ | 1.00 | 0.31^**^ |
| Wall thickness (mm) | 0.04 | 0.40^**^ | 0.31^**^ | 1.00 |

**, significant at *P* < 0.01.

**Supplementary Table 3.** Statistic of RNA-seq, ddRAD-seq data to *R. chinensis* and the reference genome data of *S. chinensis*.

| Transcriptome of *R. chinensis* | | Reduced-representation genome of *R. chinensis* | | Reference genome of *S. chinensis* | |
| --- | --- | --- | --- | --- | --- |
| Raw reads | 43,987,994 | Raw reads | 22,873,680 | Total number of sequences examined | 208 |
| Nucleotides of raw reads | 6,598,199,100 | Nucleotides of raw reads | 2,946,230,820 | Total size of examined sequences (bp) | 280427550 |
| Clean reads | 43,987,592 | Clean reads | 22,872,914 | Total number of identified SSRs | 338031 |
| Nucleotides of clean reads | 6,598,138,800 | Nucleotides of clean reads | 2,946,085,731 | Number of SSR containing sequences | 202 |
| GC content of clean reads | 45.12 | GC content of clean reads | 41 |  |  |
| Unigenes | 34,976 | Contigs | 69,134 |  |  |
| Average length of unigenes | 990.47 | Average length of contigs | 88.46 |  |  |
| N50 of unigenes | 1766 | Unitigs | 3,438,148 |  |  |
| SSRs identified from unigenes | 6958 | Average length of unitigs | 46.22 |  |  |
| Unigenes containing SSRs | 5513 | SSRs identified from contigs and unitigs | 53,004 |  |  |
|  |  | Sequence containing SSRs | 51,937 |  |  |

**Supplementary Table 4.** Genetic characterization of 23 pairs of *R. chinensis* SSR primers and 9 pairs of *S. chinensis* SSR primers.

| Locus ­Name | Primer Sequence (5'-3') | Repeat Motif | Product Size (bp) | *N*_A_ | *N*_E_ | *H*_O_ | *H*_E_ | *PIC* | *I* | *H* | *F*_ST_ | *N*_m_ |
| --- | --- | --- | --- | --- | --- | --- | --- | --- | --- | --- | --- | --- |
| *Rc*-eSSR01 | F: TTGTAGTGGTGAGGATTGCG | (AAG)7 | 185 | 5 | 2.33 | 0.50 | 0.57 | 0.48 | 0.99 | 0.57 | 0.13 | 1.74 |
|  | R: GCCACGCTTCCACGTATTTA |  |  |  |  |  |  |  |  |  |  |  |
| *Rc*-eSSR16 | F: ATAACCAAGGCAGCAGCATC | (AGA)8 | 153 | 6 | 2.88 | 0.61 | 0.66 | 0.61 | 1.31 | 0.65 | 0.06 | 3.68 |
|  | R: TTCACCTCTTCCCTTCCCTT |  |  |  |  |  |  |  |  |  |  |  |
| *Rc*-eSSR23 | F: AGCCCTTTTTGCCTTCTCTC | (AAG)6 | 128 | 6 | 2.53 | 0.59 | 0.61 | 0.55 | 1.12 | 0.61 | 0.05 | 4.6 |
|  | R: TCTCTCTCAGCCCTTAAACC |  |  |  |  |  |  |  |  |  |  |  |
| *Rc*-eSSR26 | F: TTCCCATGCTATTCCACCTC | (TCT)5 | 191 | 5 | 2.53 | 0.56 | 0.61 | 0.54 | 1.1 | 0.61 | 0.17 | 1.25 |
|  | R: ACGTGGAACTGCTTTGTGG |  |  |  |  |  |  |  |  |  |  |  |
| *Rc*-eSSR34 | F: TTTCCTCCGTCGTCTTCACT | (CTT)10 | 187 | 5 | 2.38 | 0.58 | 0.58 | 0.51 | 1.01 | 0.58 | 0.09 | 2.66 |
|  | R: TCTGTGTCGTCAGTTCAGCC |  |  |  |  |  |  |  |  |  |  |  |
| *Rc*-eSSR39 | F: TTTAGCATACCCGAAATCCG | (GGT)5 | 158 | 2 | 1.5 | 0.34 | 0.33 | 0.28 | 0.52 | 0.33 | 0.08 | 2.89 |
|  | R: GCGTTCAAGGAAGGAATGA |  |  |  |  |  |  |  |  |  |  |  |
| *Rc*-eSSR41 | F: AGCGGGACTAGGGTTTGTTT | (GAA)7 | 169 | 9 | 5.71 | 0.82 | 0.83 | 0.8 | 1.88 | 0.83 | 0.06 | 3.73 |
|  | R: GAGCTCCACAACGACTCTC |  |  |  |  |  |  |  |  |  |  |  |
| *Rc*-eSSR50 | F: GGCCGATGAGGAACAATAGA | (GA)7 | 157 | 11 | 2.87 | 0.70 | 0.66 | 0.64 | 1.57 | 0.65 | 0.09 | 2.42 |
|  | R: GTGCCACCCAGAATCAGAC |  |  |  |  |  |  |  |  |  |  |  |
| *Rc*-eSSR51 | F: CCAGTAGCATGCGAGGAAAT | (AT)8 | 238 | 9 | 3.75 | 0.74 | 0.74 | 0.69 | 1.55 | 0.73 | 0.05 | 4.59 |
|  | R: GTCATTGCTGGGAGGTGTTT |  |  |  |  |  |  |  |  |  |  |  |
| *Rc*-eSSR53 | F: TTGCAGTACGTGAAGGATGC | (TA)6 | 265 | 6 | 1.35 | 0.24 | 0.26 | 0.25 | 0.61 | 0.26 | 0.05 | 4.32 |
|  | R: CATGAATGGAGTGTTAGGG |  |  |  |  |  |  |  |  |  |  |  |
| *Rc*-eSSR55 | F: CCGTTCAACACTTGGGTTCT | (GA)10 | 144 | 5 | 2.33 | 0.55 | 0.57 | 0.5 | 1.03 | 0.57 | 0.06 | 4.14 |
|  | R: TTCCGTTTCGTCTTCACACA |  |  |  |  |  |  |  |  |  |  |  |
| *Rc*-eSSR58 | F: CTGAGGAGCACCCTTAGGAA | (AG)11 | 277 | 10 | 2.15 | 0.50 | 0.54 | 0.51 | 1.18 | 0.54 | 0.03 | 7.74 |
|  | R: AAACACAGACACGTCTCCC |  |  |  |  |  |  |  |  |  |  |  |
| *Rc*-gSSR74 | F: TGGCAAGAAGAGTAAATGTGAAA | (TA)8 | 139 | 12 | 2.26 | 0.62 | 0.56 | 0.52 | 1.23 | 0.56 | 0.08 | 2.73 |
|  | R: CGAGCGGCTTGACTCAATG |  |  |  |  |  |  |  |  |  |  |  |
| *Rc*-gSSR86 | F: GATGAGGACAGCTGCAGACA | (AT)6 | 157 | 9 | 3.21 | 0.54 | 0.69 | 0.62 | 1.31 | 0.69 | 0.08 | 2.75 |
|  | R: GGCTCTGATACACCTTCCGA |  |  |  |  |  |  |  |  |  |  |  |
| *Rc*-gSSR87 | F: CCCAAAGTTCACACTTCAAACC | (TA)6 | 137 | 4 | 1.84 | 0.45 | 0.46 | 0.37 | 0.73 | 0.46 | 0.05 | 4.42 |
|  | R: TAGTTGCTTTCAGGGCACA |  |  |  |  |  |  |  |  |  |  |  |
| *Rc*-gSSR90 | F: CTGAATGAGTCAGTAAGGGGAT | (AT)7 | 115 | 7 | 2.65 | 0.06 | 0.63 | 0.58 | 1.3 | 0.62 | 0.21 | 0.95 |
|  | R: GAAATTCTCGTTCCCGATTG |  |  |  |  |  |  |  |  |  |  |  |
| *Rc*-gSSR92 | F: TGGGCAGAAACATCTATTACCA | (TA)10 | 108 | 13 | 4.39 | 0.38 | 0.78 | 0.77 | 1.89 | 0.77 | 0.18 | 1.1 |
|  | R: GCATGTCGCTTGAACAGTTT |  |  |  |  |  |  |  |  |  |  |  |
| *Rc*-gSSR97 | F: GAACCCAAAAAGGCTAACCC | (CTC)5 | 125 | 5 | 1.95 | 0.5 | 0.49 | 0.44 | 0.92 | 0.49 | 0.05 | 4.6 |
|  | R: TGAGGAAGCAAAAGAGCA |  |  |  |  |  |  |  |  |  |  |  |
| *Rc*-gSSR100 | F: AGATCGATAATGGCCGTGG | (AGA)5 | 128 | 4 | 1.36 | 0.27 | 0.27 | 0.25 | 0.53 | 0.27 | 0.1 | 2.34 |
|  | R: CCATTGAGGCAGACCCTAA |  |  |  |  |  |  |  |  |  |  |  |
| *Rc*-gSSR119 | F: GCCCTCAAATCCACCACTAA | (CAC)6 | 135 | 3 | 2.02 | 0.52 | 0.51 | 0.39 | 0.75 | 0.51 | 0.1 | 2.36 |
|  | R: TAACTTCAAGGAGCCATCG |  |  |  |  |  |  |  |  |  |  |  |
| *Rc*-gSSR120 | F: AACAATGTTCGCTACCAGGC | (CTG)5 | 150 | 4 | 1.97 | 0.50 | 0.49 | 0.38 | 0.73 | 0.49 | 0.03 | 7.27 |
|  | R: AGTAAGAGCTCACCACCCA |  |  |  |  |  |  |  |  |  |  |  |
| *Rc*-gSSR135 | F: ATGCCTGCTGGAATGAAAAC | (AGG)5 | 151 | 6 | 1.52 | 0.21 | 0.35 | 0.32 | 0.71 | 0.34 | 0.09 | 2.64 |
|  | R: ACCTTGTCCCATTTCTGTGC |  |  |  |  |  |  |  |  |  |  |  |
| *Rc*-gSSR137 | F: TGTTTGAGACTGAGTCAATGAAATC | (AAT)7 | 106 | 5 | 2.65 | 0.57 | 0.63 | 0.55 | 1.15 | 0.62 | 0.12 | 1.78 |
|  | R: ATCCCTACGCTGACCACAG |  |  |  |  |  |  |  |  |  |  |  |
| *Rc*-eSSRmean |  |  | 188 | 6.58 | 2.69 | 0.56 | 0.58 | 0.53 | 1.16 | 0.58 | 0.08 | 3.65 |
| *Rc*-gSSRmean |  |  | 132 | 6.55 | 2.35 | 0.42 | 0.53 | 0.47 | 1.02 | 0.53 | 0.1 | 2.99 |
| *Rc*-Mean |  |  | 161 | 6.57 | 2.53 | 0.49 | 0.56 | 0.5 | 1.09 | 0.55 | 0.09 | 3.33 |
| *Sc*-gSSR01 | F: CGTCGTCAGCCGCCTCAC | (CGT)5 | 120 | 5 | 1.66 | 0.36 | 0.4 | 0.37 | 0.8 | 0.4 | 0.06 | 3.77 |
|  | R: GGCCGCCGCGCTCTTTAA |  |  |  |  |  |  |  |  |  |  |  |
| *Sc*-gSSR02 | F: TCACAAAACAACAGACCG | (TTTG)5 | 298 | 6 | 3.12 | 0.46 | 0.68 | 0.62 | 1.29 | 0.68 | 0.06 | 3.83 |
|  | R: AGAACAATTATTCAAGCG |  |  |  |  |  |  |  |  |  |  |  |
| *Sc*-gSSR03 | F: TACCCTTTCTAACTGCTT | (ATA)9 | 249 | 8 | 3.65 | 0.60 | 0.73 | 0.69 | 1.58 | 0.73 | 0.03 | 7.37 |
|  | R: CTCTTTCACTCTCTCTGC |  |  |  |  |  |  |  |  |  |  |  |
| *Sc*-gSSR04 | F: CCGTATGCTTTTCTTTTT | (GCC)6 | 188 | 9 | 2.56 | 0.54 | 0.61 | 0.58 | 1.3 | 0.61 | 0.04 | 6.09 |
|  | R: GTCGACTTGATCGGTTTT |  |  |  |  |  |  |  |  |  |  |  |
| *Sc*-gSSR06 | F: TTTCTTACGATTTATGCG | (TG)9 | 293 | 16 | 7.03 | 0.59 | 0.86 | 0.84 | 2.17 | 0.86 | 0.05 | 4.55 |
|  | R: CGAGAGAGACGTGCTCTC |  |  |  |  |  |  |  |  |  |  |  |
| *Sc*-gSSR07 | F: GTCCACCGTGCGGATTAT | (AC)12 | 177 | 16 | 7.49 | 0.68 | 0.87 | 0.85 | 2.3 | 0.87 | 0.06 | 3.6 |
|  | R: ACTGTTGCCGTTGCTATT |  |  |  |  |  |  |  |  |  |  |  |
| *Sc*-gSSR08 | F: GAGCCCGATGTAAACAAG | (CCG)8 | 155 | 9 | 3.87 | 0.70 | 0.75 | 0.71 | 1.59 | 0.74 | 0.05 | 5.08 |
|  | R: TACGACGCTATGGAAAAA |  |  |  |  |  |  |  |  |  |  |  |
| *Sc*-gSSR09 | F: ACTGTCTCGGCACGGCTG | (GC)6 | 225 | 6 | 2.51 | 0.58 | 0.6 | 0.54 | 1.11 | 0.6 | 0.04 | 5.52 |
|  | R: CGGTTTTCGTTTATGTTT |  |  |  |  |  |  |  |  |  |  |  |
| *Sc*-gSSR10 | F: CAAACGCAAGAACTTGATT | (CGG)6 | 222 | 10 | 6.19 | 0.82 | 0.84 | 0.82 | 1.98 | 0.84 | 0.04 | 6.08 |
|  | R: CTGTCGCACAGGTGACACT |  |  |  |  |  |  |  |  |  |  |  |
| *Sc*-Mean |  |  | 214 | 9.44 | 4.23 | 0.59 | 0.71 | 0.67 | 1.57 | 0.7 | 0.05 | 4.86 |

**Supplementary Table 5.** Analysis of molecular variance (AMOVA) for *R. chinensis* and *S. chinensis* populations.

| Species | Source | Degrees of freedom | Sum of square | Mean of square | Variance components | Percentage of variation |
| --- | --- | --- | --- | --- | --- | --- |
| *R. chinensis* | Among Populations | 9 | 127.55 | 14.17 | 0.36 | 6% |
|  | Among Individuals | 92 | 631.21 | 6.86 | 0.67 | 10% |
|  | Within Individuals | 102 | 563.00 | 5.52 | 5.52 | 84% |
|  | Total | 203 | 1321.76 |  | 6.55 | 100% |
| *S. chinensis* | Among Populations | 9 | 31.43 | 3.49 | 0.00 | 0% |
|  | Among Individuals | 92 | 334.96 | 3.64 | 0.51 | 16% |
|  | Within Individuals | 102 | 268.00 | 2.63 | 2.63 | 84% |
|  | Total | 203 | 634.39 |  | 3.13 | 100% |

**Supplementary Table 6.** Sampling information.

| Population | Location | Growth pattern | Longitude° | Latitude° | Sample size |
| --- | --- | --- | --- | --- | --- |
| WJP-W | Wang Jiaping | Wild | 111.02 | 30.24 | 10 |
| ­WJP-C | Wang Jiaping | Cultivated | 111.02 | 30.24 | 9 |
| BNG-W | Bai Nianguan | Wild | 110.95 | 30.18 | 10 |
| BNG-C | Bai Nianguan | Cultivated | 110.95 | 30.18 | 10 |
| BLZ-W | Bai Luzhuang | Wild | 110.73 | 30.19 | 11 |
| BLZ-C | Bai Luzhuang | Cultivated | 110.73 | 30.19 | 10 |
| HLP-W | Huang Liangping | Wild | 110.66 | 30.29 | 11 |
| HLP-C | Huang Liangping | Cultivated | 110.66 | 30.29 | 11 |
| HS-W | Huo Shan | Wild | 110.44 | 30.30 | 10 |
| HS-C | Huo Shan | Cultivated | 110.44 | 30.30 | 10 |

**Supplementary Table 7.** Method validation of the high-performance liquid chromatography (HPLC) analysis in horned galls.

| Compound | Gallic acid |
| --- | --- |
| Calibration curve | y=57805x - 39520 |
| *R*^2^ | 0.9993 |
| Linear range (ug/ml) | 6.375 - 102 |
| Precision (RSD, %) | 1.65 |
| Repeatability (RSD, %) | 2.31 |
| Stability (RSD, %) | 1.83 |
| Extraction recovery (%) | 96.82 |
